# Comprehensive assessment of transcriptome assembly quality using CATS

**DOI:** 10.1038/s41467-026-72171-8

**Published:** 2026-04-20

**Authors:** Kristian Bodulić, Kristian Vlahoviček

**Affiliations:** 1 Department for Bioinformatics and Statistics, University Hospital for Infectious Diseases “Dr. Fran Mihaljević”, Zagreb, Croatia; 2 Bioinformatics Group, Division of Molecular Biology, Department of Biology, Faculty of Science, University of Zagreb, Zagreb, Croatia

**Keywords:** Software, Genome informatics, Transcriptomics

## Abstract

Accurate assessment of transcriptome assembly quality is critical to ensure the reliability of subsequent transcriptomic analyses. Here, we present CATS (Comprehensive Assessment of Transcript Sequences), a tool offering both reference-free (CATS-rf) and reference-based (CATS-rb) transcriptome quality evaluation pipelines. CATS-rf maps RNA-seq reads back to the assembled transcripts and computes four interpretable scoring components that capture common assembly errors. CATS-rb assesses transcriptome completeness via alignment to a reference genome, supporting both annotation-free and annotation-based scoring. We benchmarked CATS on 1056 transcriptomes from simulated and public RNA-seq data. CATS-rf outperformed existing tools in transcript-level accuracy assessment and demonstrated high sensitivity to diverse assembly error types. CATS-rb produced robust transcriptome completeness estimates even without external annotation, with its scoring metrics strongly reflecting assembly quality. These results highlight CATS as an accurate, interpretable, and broadly applicable framework for evaluating transcriptome assemblies.

## Abstract (web summary)

Accurate assessment of transcriptome assembly quality is essential for reliable downstream analyses. Here, the authors present CATS, an interpretable framework offering both reference-free and reference-based transcriptome assembly evaluation with detailed quality reporting.

## Introduction

Accurate assembly of RNA-seq data into full-length transcripts is a prerequisite to virtually any downstream analysis procedure^1,2^. Most transcriptome assemblers build de Bruijn graphs from read k-mers and reconstruct transcripts by traversing the graph paths^1–6^. However, the assembly process is challenged by features intrinsic to transcriptomic data, including non-uniform coverage and alternative splicing. These issues are pronounced in low-abundance transcripts, which are frequently misassembled. De novo assemblers implement various approaches to mitigate these challenges, including read correction and normalization, multi-k-mer strategies, and clustering of isoforms into subgraphs^3–6^. Benchmarking studies report considerable variability in assembly quality driven by transcriptome complexity, sequencing depth, library quality, and assembler choice. The observed variability is exacerbated by the large parameter space among assemblers, further complicating the generalization of optimal assembly strategies^7–12^. These challenges underscore the need for reliable methods which assess transcriptome assembly quality and precisely characterize detected assembly errors.

Transcriptome quality assessment tools are broadly categorized by their use of reference data as reference-free or reference-based. RSEM-EVAL and TransRate are the only tools specifically performing reference-free transcriptome quality evaluation. RSEM-EVAL employs a probabilistic model that considers the joint likelihood of the assembly and the RNA-seq reads, producing a global assembly score that incorporates the quality of assembled transcripts and assembly completeness relative to the input RNA-seq library. The tool also reports transcript-level metrics which measure the contribution of each transcript to the overall assembly score. By definition, these transcript scores do not directly reflect transcript quality and are difficult to interpret in terms of specific error types. The performance of RSEM-EVAL is further constrained by several limitations. For instance, RSEM-EVAL’s penalty against assembly complexity may favor overly simplistic assemblies. The tool is also permissive of assemblies that merge low-coverage transcripts, leading to inflated scores for structurally implausible reconstructions. Furthermore, RSEM-EVAL’s alignment algorithm does not account for indels, reducing sensitivity to certain types of errors^13^.

TransRate addresses some of these limitations by estimating assembly quality directly from read alignment evidence. The tool maps RNA-seq reads to assembled transcripts and uses transcript abundance to probabilistically assign multimapped reads. Transcript scores are inferred based on deviations from expected mapping patterns, thereby providing evidence of transcript quality by detecting distinct error types. TransRate integrates these transcript-level metrics with the proportion of RNA-seq reads that successfully map to the assembly to compute a global assembly score capturing both transcript accuracy and assembly completeness. Despite the alignment–based error detection, the tool suffers from several limitations. In particular, TransRate often underestimates the quality of lowly expressed isoforms. Another drawback is the independent assignment of multimapped paired-reads, introducing artificial penalties for isoforms and paralogues. TransRate also poorly distinguishes alignment errors caused by low read quality or transcript variation from mapping inconsistencies resulting from misassembly^14^. Additionally, the tool appears to be no longer maintained^15^.

RNAQuast and SQANTI3 are widely used reference-based pipelines for transcriptome quality assessment, leveraging reference genomes to produce alignment-based and completeness metrics. Both tools rely on genomic annotations, limiting their ability to assess the fidelity of unannotated transcripts. Furthermore, completeness metrics are calculated at the isoform level, potentially introducing bias by including transcript models with overlapping exon structures. SQANTI3 is specifically designed for long-read sequencing data, making it unsuitable for evaluating short-read or hybrid transcriptome assemblies. Notably, neither tool provides a unified score reflecting overall assembly completeness, hindering direct assembly-to-assembly comparison^16,17^.

Here, we introduce CATS (Comprehensive Assessment of Transcript Sequences), a framework for transcriptome quality evaluation. CATS features both reference-free (CATS-rf) and reference-based (CATS-rb) pipelines, designed to alleviate the limitations of existing methods. Both tools provide assembly quality scores complemented by a comprehensive set of supporting metrics. Through extensive testing on simulated and public datasets, we demonstrate that CATS is a robust and accurate solution for transcriptome assembly assessment.

## Results

### Overview of the CATS-rf pipeline

**Fig. 1:**
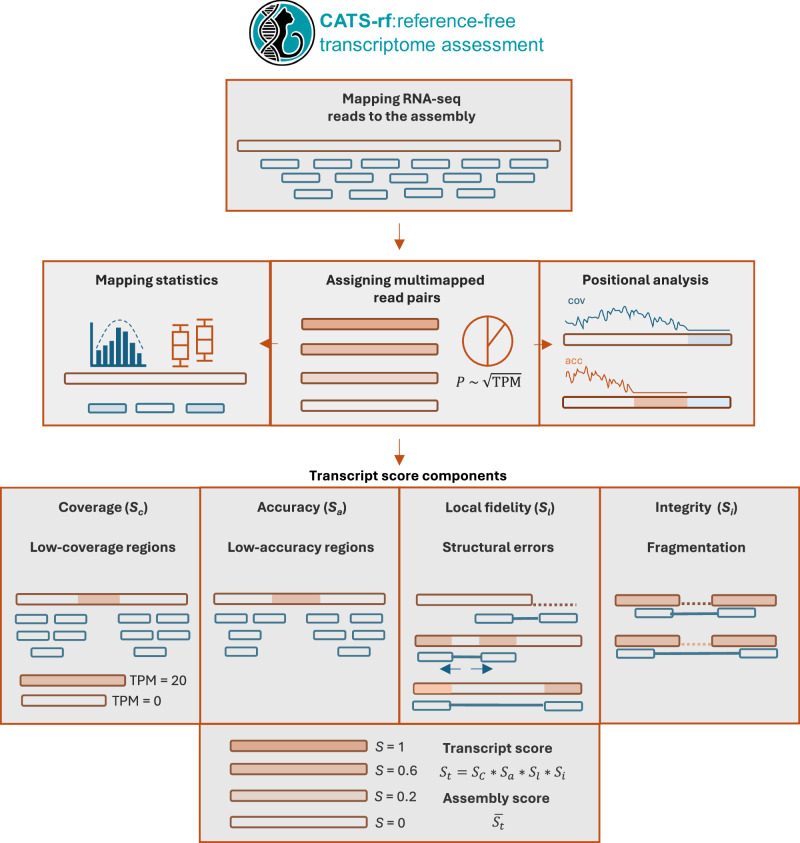
Overview of transcriptome assembly evaluation by CATS-rf. RNA-seq reads are mapped back to assembled transcripts, with multimapped reads probabilistically assigned based on transcript abundance. Read alignment data are used to compute four transcript-level score components: coverage, accuracy, local fidelity, and integrity. Each component targets a specific class of assembly errors, including insertions, mismatches, structural inconsistencies, and fragmentation. Transcript scores are calculated as the product of the four components, with the assembly score defined as the mean of individual transcript scores. *P* probability of read assignment, TPM transcripts per million, *S*_*t*_ transcript score, *S*_*c*_ coverage component, *S*_*a*_ accuracy component, *S*_*l*_ local fidelity component, *S*_*i*_ integrity component.

CATS-rf (reference-free) evaluates transcriptome assembly quality using short-read RNA-seq data employed in the assembly process (Fig. 1). The pipeline maps reads to the analyzed transcripts, assigning multimapped reads probabilistically based on transcript abundance. Assembly evaluation is performed at the transcript level, integrating four score components each targeting specific assembly errors. The coverage component flags low-coverage regions suggesting unsupported insertions or redundancy, while the accuracy component detects segments exhibiting sequence inaccuracy. Both components apply penalties proportional to the length of affected regions. The local fidelity component captures inconsistent paired-read mapping within individual transcripts, suggesting structural errors such as deletions, inversions, or translocations. These inconsistencies include reads with unmapped pairs, inaccurate mapping orientations, or abnormal insert sizes. The integrity component identifies transcript fragmentation through read pairs mapping to separate transcripts, with penalties scaled based on their proximity to transcript ends. Transcript quality score is defined as the product of the four components, with the assembly quality score calculated as the mean of individual transcript scores. Accordingly, CATS-rf scores exclusively capture the quality of assembled transcripts.

### Association of CATS-rf scores with assembly features and quality

**Fig. 2:**
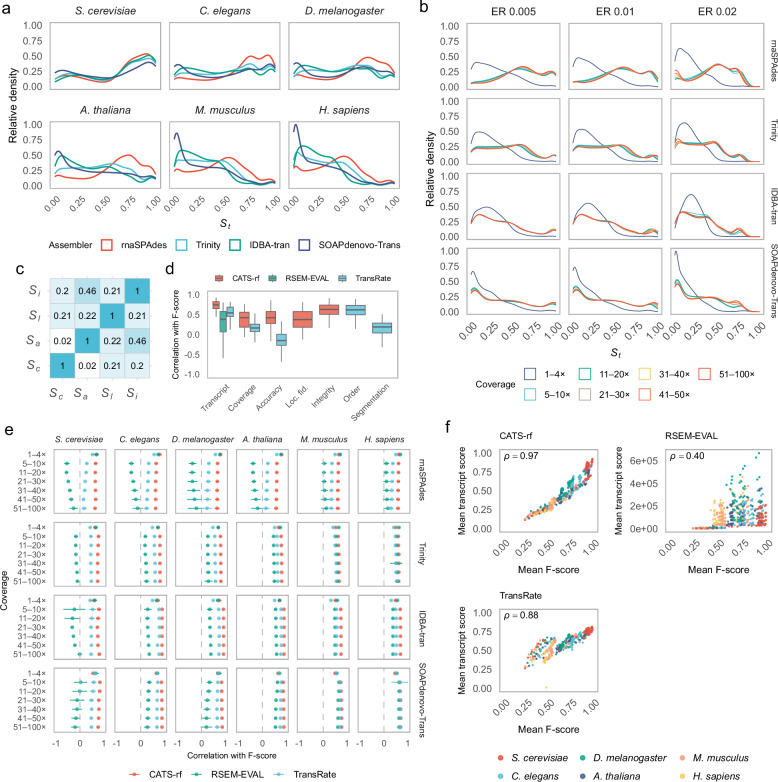
Association of CATS-rf scores with assembly features and quality in controlled simulations. **a** Distribution of CATS-rf transcript scores across species and transcriptome assemblers. Colors indicate assemblers: red = rnaSPAdes, light blue = Trinity, green = IDBA-tran, dark blue = SOAPdenovo-Trans. **b** Distribution of CATS-rf transcript scores by transcript coverage, sequencing error rate, and assembler. Colors indicate coverage: dark blue = 1–4×, light blue = 5–10×, green = 11–20×, brown = 21–30×, yellow = 31–40×, orange = 41–50×, red = 51–100×. **c** Median pairwise Spearman correlation coefficients between CATS-rf score components across *n* = 504 assemblies. **d** Distribution of Spearman correlation coefficients between transcript F-scores and transcript scores from CATS-rf, RSEM-EVAL, and TransRate across *n* = 504 assemblies. Boxplots represent the median and IQR, with whiskers extending to ±1.5 × IQR. **e** Spearman correlation coefficients of transcript scores from all three tools with transcript F-scores across species, coverage levels, and assemblers. Each point represents the median across *n* = 3 assemblies corresponding to libraries with distinct sequencing error rates, with error bars indicating the IQR. In (**d**) and (**e**), colors indicate transcriptome quality assessment tools: red = CATS-rf, green = RSEM-EVAL, light blue = TransRate. **f** Correlation of mean transcript scores from CATS-rf, RSEM-EVAL, and TransRate with mean transcript F-scores across assemblies. Colors represent species: red *Saccharomyces cerevisiae*, light blue *Caenorhabditis elegans*, green = *Drosophila melanogaster*, dark blue = *Arabidopsis thaliana*, salmon = *Mus musculus*, yellow = *Homo sapiens. S*_*t*_ = transcript score, ER = error rate, *S*_*c*_ = coverage component, *S*_*a*_ = accuracy component, *S*_*l*_ = local fidelity component, *S*_*i*_ = integrity component, *ρ* = Spearman correlation coefficient.

CATS-rf evaluation was performed on a comprehensive dataset consisting of simulated and public RNA-seq libraries. We first designed an orthogonal simulation experiment encompassing transcriptomes assembled from 126 simulated libraries, covering six model species, seven transcript coverage ranges, and three sequencing error rates (Fig. 2). Each library was assembled with four relevant de novo transcriptome assemblers. This controlled framework featured fixed coverage levels and complete representation of reference transcripts, allowing us to isolate the effects of transcriptome complexity, coverage, sequencing error rate, and assembler choice on CATS scoring.

The distribution of CATS-rf transcript scores varied significantly across species and assemblers (Fig. 2a). Transcriptomes with higher complexity, such as those of *M. musculus and H. sapiens*, yielded lower transcript scores, with rnaSPAdes and Trinity consistently outperforming IDBA-tran and SOAPdenovo-Trans. Low coverage levels led to significantly reduced transcript scores across all assemblies (Fig. 2b). Increasing coverage beyond 5–10× generally yielded marginal improvements to transcript scores, although the gains were more evident in libraries with higher sequencing error rates.

To assess redundancy among CATS-rf score components, we calculated their pairwise correlations within each assembly and averaged them across assemblies. Score components showed weak to moderate intercorrelations, with median *ρ* = 0.02 (IQR = 0.24) to 0.46 (IQR = 0.20) (Fig. 2c). This finding was consistent across species, coverage levels, and assemblers, indicating that each component captures distinct aspects of assembly quality (Supplementary Fig. 1).

We first benchmarked CATS-rf against existing reference-free transcriptome assessment tools using the controlled dataset, enabling evaluation under idealized conditions featuring a broad range of assembly characteristics. TransRate and CATS-rf directly evaluate individual transcript quality, whereas the RSEM-EVAL transcript score reflects each transcript’s contribution to the overall assembly score and is expected to correlate with transcript quality within the same dataset. As such, transcript scores from CATS-rf, RSEM-EVAL, and TransRate were correlated with transcript F-scores derived from reciprocal best hits to reference transcripts, serving as an objective measure of accuracy. The stated correlations were first calculated within each assembly (Fig. 2d). CATS-rf transcript scores exhibited the strongest association with F-scores (median *ρ* = 0.75, IQR = 0.17), outperforming RSEM-EVAL (median *ρ* = 0.38, IQR = 0.51) and TransRate (median *ρ* = 0.46, IQR = 0.23). The CATS-rf coverage component correlated more strongly with F-scores (median *ρ* = 0.42, IQR = 0.37) than the TransRate equivalent (median *ρ* = 0.16, IQR = 0.18). Similarly, the CATS-rf accuracy component (median *ρ* = 0.42, IQR = 0.30) outperformed its TransRate counterpart (median *ρ* = −0.15, IQR = 0.28). While TransRate aggregates evidence for structural errors and fragmentation into a single metric (the order score), CATS-rf separates these into distinct components, both showing moderate correlations with F-scores (local fidelity: median *ρ* = 0.38, IQR = 0.40, integrity: median *ρ* = 0.63, IQR = 0.23).

In-depth benchmarking showed that CATS-rf consistently outperformed both RSEM-EVAL and TransRate across all analyzed taxa, assemblers, and non-minimum coverage levels (Fig. 2e, Supplementary Fig. 2). The largest performance gains were found in rnaSPAdes assemblies. At the lowest coverage level (1-4×), CATS-rf and RSEM-EVAL exhibited similar accuracy, slightly outperforming TransRate.

Additionally, we assessed the association between mean transcript quality scores produced by each evaluation tool and mean transcript F-scores across assemblies (Fig. 2f). This analysis examined the ability of transcript quality metrics to rank datasets according to the absolute accuracy of assembled transcripts across heterogeneous libraries. CATS-rf again outperformed its counterparts, showing stronger correlation with mean F-scores (*ρ* = 0.97, 95%CI 0.96–0.97) compared to RSEM-EVAL (*ρ* = 0.40, 95%CI 0.32–0.47, z = 28.25, *p* < 0.001) and TransRate (*ρ* = 0.88, 95%CI 0.88–0.88, z = 14.24, *p* < 0.001).

**Fig. 3:**
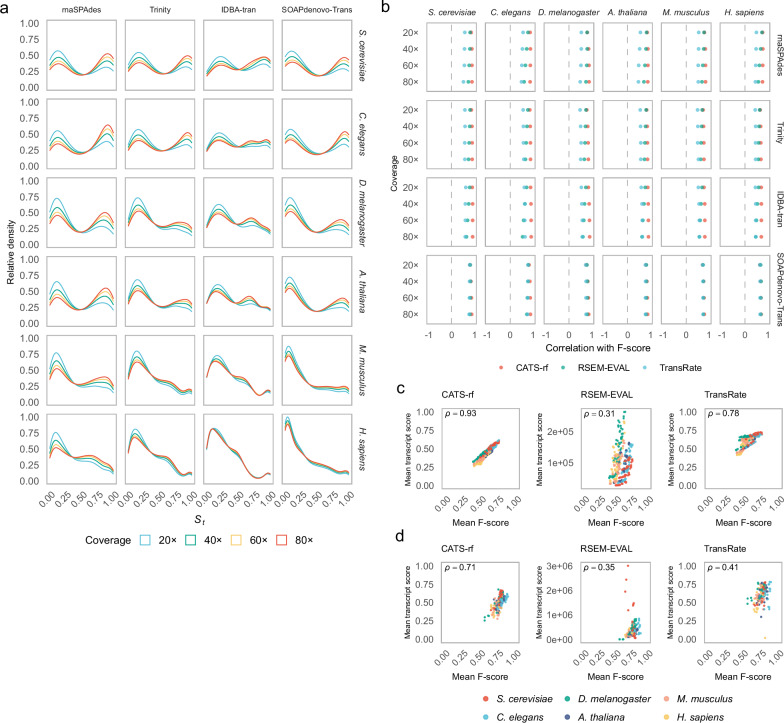
Association of CATS-rf scores with assembly features and quality in realistically simulated and public datasets. **a** Distribution of CATS-rf transcript scores across species, assemblers, and sequencing coverage in realistically simulated datasets. Colors indicate coverage: light blue = 20×, green = 40×, yellow = 60×, red = 80×. **b** Spearman correlation coefficients of transcript scores from CATS-rf, RSEM-EVAL, and TransRate with transcript F-scores across assemblies from realistically simulated libraries. Plotted values represent the median across *n* = 4 library replicates for each species and sequencing depth combination, with error bars omitted due to minimal variability across replicates (IQR < 0.03). Colors indicate transcriptome quality assessment tools: red = CATS-rf, green = RSEM-EVAL, light blue = TransRate. **c** Correlation of mean transcript scores from CATS-rf, RSEM-EVAL, and TransRate with mean transcript F-scores across realistically simulated assemblies. **d** Equivalent correlations to (**c**) for the publicly available dataset. In (**c**) and (**d**), colors represent species: red = *Saccharomyces cerevisiae*, light blue =*Caenorhabditis elegans*, green = *Drosophila melanogaster*, dark blue = *Arabidopsis thaliana*, salmon = *Mus musculus*, yellow = *Homo sapiens*. *S*_*t*_ = transcript score, *ρ* = Spearman correlation coefficient.

Next, we analyzed CATS-rf scoring under realistic conditions (Fig. 3). This first included simulated realistic datasets exhibiting multifold intra-library variation in coverage and a lower proportion of expressed transcripts. The setup comprised 96 libraries spanning the same six species and four coverage levels, with four replicates per combination. Each library was assembled using the same suite of tools, resulting in 384 assemblies. The transcript score distributions reflected an intermediate of low- and higher-coverage profiles, consistent with the heterogeneous depth of realistic libraries (Fig. 3a). Nonetheless, CATS-rf scores remained positively correlated with sequencing depth, and complex transcriptomes generally exhibited lower quality. Regarding assemblers, rnaSPAdes and Trinity again showed the strongest performance. CATS-rf achieved higher correlation between transcript scores and F-scores within assemblies than RSEM-EVAL and TransRate (CATS-rf: median *ρ* = 0.85, IQR = 0.10, RSEM-EVAL: median *ρ* = 0.73, IQR = 0.12, TransRate: median *ρ* = 0.57, IQR = 0.13). Moreover, CATS-rf outperformed its competitors in virtually all datasets, excluding six SOAPdenovo-Trans assemblies with the lowest coverage (Fig. 3b). Mean CATS-rf transcript scores also exhibited a stronger correlation with mean transcript F-scores (*ρ* = 0.93, 95%CI 0.92–0.95) compared to transcript accuracy estimates from RSEM-EVAL (*ρ* = 0.31, 95%CI 0.21–0.40, z = 20.17, *p* < 0.001) and TransRate (*ρ* = 0.78, 95%CI 0.74–0.82, z = 11.11, *p* < 0.001, Fig. 3c).

We also applied CATS-rf to 168 transcriptomes assembled from 42 public RNA-seq libraries. CATS-rf transcript scores were overall lower than those from simulated assemblies (Supplementary Fig. 3). Consistent with simulated data, complex transcriptomes generally yielded lower scores. Among assemblers, rnaSPAdes and Trinity exhibited the highest scores. Comparative benchmark was performed on transcripts present in the corresponding reference transcriptomes (Fig. 3d). CATS-rf demonstrated the strongest correlation between mean transcript scores and mean F-scores (*ρ* = 0.71, 95%CI 0.62–0.78 compared to RSEM-EVAL (*ρ* = 0.35, 95%CI 0.21–0.47, z = 5.14, *p* < 0.001) and TransRate (*ρ* = 0.41, 95%CI 0.28–0.53, z = 4.51, *p* < 0.001).

### Association between CATS-rf scores and assembly errors

**Fig. 4:**
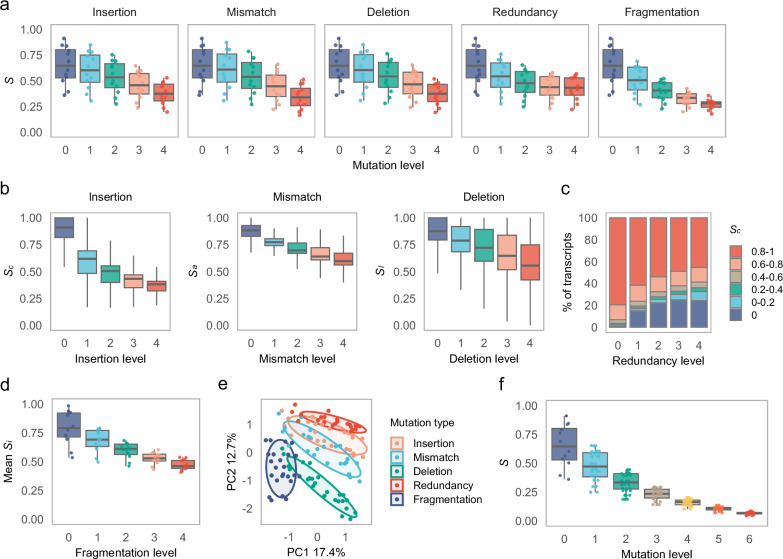
Association between CATS-rf scores and assembly errors. **a** Distribution of assembly scores across *n* = 12 assemblies per mutation level with increasing levels of insertions, mismatches, deletions, redundancy, and fragmentation. **b** Distribution of coverage, accuracy, and local fidelity score components in transcripts with increasing levels of insertions, mismatches, and deletions, respectively (*n* = 2019326, 121152, 242311, 363468, and 484627 transcripts per respective mutation level). **c** Distribution of the coverage score component across transcripts with increasing redundancy. Values are binned and color-coded as follows: dark blue = 0, light blue = 0–0.2, green = 0.2–0.4, brown = 0.4–0.6, salmon = 0.6–0.8, red = 0.8–1. **d** Distribution of the mean integrity score component across *n* = 12 assemblies per fragmentation level. In (**a**), (**b**), and (**d**), color denotes mutation levels: dark blue = 0, light blue = 1, green = 2, salmon = 3, red = 4. **e** Principal component analysis of mean transcript score components in assemblies with distinct mutation types. Colors indicate mutation type: dark blue = fragmentation, red = redundancy, green = deletion, light blue = mismatch, salmon = insertion. **f** Distribution of assembly scores in assemblies with increasing levels of multiplicative mutations. Mutation level 0 corresponds to *n* = 12 native assemblies, while levels 1–6 each encompass *n* = 36 assemblies. Color denotes mutation levels: dark blue = 0, light blue = 1, green = 2, brown = 3, yellow = 4, orange = 5, red = 6. Boxplots represent the median and IQR, with whiskers extending to ±1.5 × IQR. Points denote individual assemblies. *S* = assembly score, *S*_*c*_ = coverage component, *S*_*a*_ = accuracy component, *S*_*l*_ = local fidelity component, *S*_*i*_ = integrity component.

Next, we directly assessed the ability of CATS-rf to detect common assembly errors (Fig. 4). We introduced four increasing levels of mutations to each assembly across a subset of 12 moderately covered assemblies from controlled simulations. This analysis aimed to isolate the effects of the analyzed mutation types on CATS-rf scoring in transcripts with sufficient coverage. Five mutation types were independently introduced: insertions, mismatches, deletions, fragmentation, and redundancy (cf. Methods). Mutated assemblies exhibited a significant decline in CATS-rf overall assembly score, proportional to the mutation level (Fig. 4a). An exception was observed in redundant assemblies, where the assembly score plateaued at the third redundancy level (60% of transcripts with 60–79% sequence duplication). We also analyzed the coverage, accuracy, and local fidelity score components of transcripts containing increasing levels of insertions, mismatches, and deletions, respectively (Fig. 4b). All three mutation types led to significant reductions in the corresponding score components, with insertions eliciting the strongest decline.

Redundant assemblies exhibited a significant reduction in the coverage component. As with the overall assembly score, this decline became less pronounced after the third redundancy level (Fig. 4c). We also recorded a gradual decline of the integrity component with increasing fragmentation (Fig. 4d). To explore the scoring patterns associated with different mutation types, we performed principal component analysis (PCA) on mean score components of assemblies with all five mutation types at mutation levels 3 and 4 (Fig. 4e). Distinct clusters emerged based on mutation type, with partial overlap between assemblies containing insertions and redundancy, as well as between assemblies with insertions and mismatches.

Finally, we evaluated the performance of CATS-rf on assemblies simultaneously incorporating all mutation types (Fig. 4f). Assembly scores substantially declined with increasing mutation intensity. Moreover, the score variance decreased at higher mutation levels.

### Overview of the CATS-rb pipeline

**Fig. 5:**
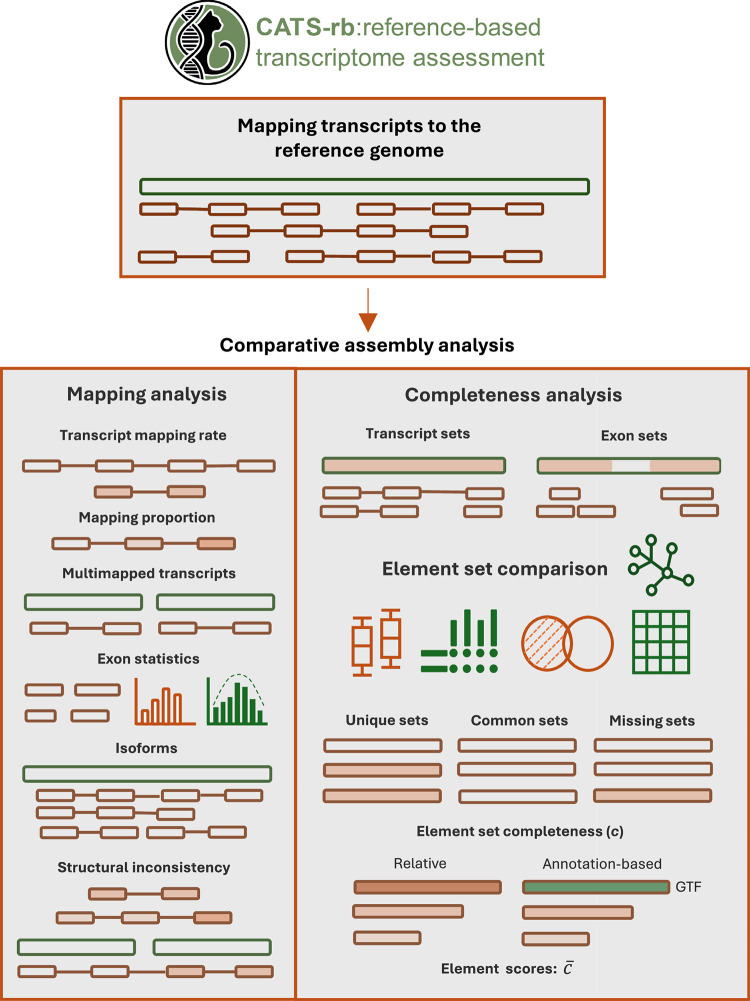
Overview of transcriptome assembly evaluation by CATS-rb. Transcripts are mapped to the reference genome and several mapping metrics are evaluated, including mappability, exon-level statistics, and structural consistency. Completeness analysis is performed across two categories of element sets, determined by overlapping transcript or exon genomic coordinates within each transcriptome assembly. Element set coordinates are overlapped between the analyzed transcriptomes, constructing a graph where edges represent element set overlaps and connected components denote element set groups. In-depth analysis of element sets is performed, visualized using UpSet plots, Venn diagrams, and hierarchical clustering heatmaps. Element set completeness is defined as the relative length of each set compared to the longest set within its group. The analysis can be extended using reference element sets derived from genomic annotation, with assembly set groups defined by shared overlaps with reference sets. Transcript and exon scores are calculated as the mean completeness of the respective element sets. *c* = Element set completeness.

CATS-rb (reference-based) evaluates transcriptome assemblies by aligning transcripts to the reference genome of the corresponding species (Fig. 5). The pipeline calculates a broad range of metrics, including transcript mappability, exon statistics, isoform composition, and structural consistency. Transcripts are classified as structurally inconsistent if they exhibit a low alignment proportion or if their segments map to disjunct genomic regions.

The core functionality of CATS-rb lies in the assessment of assembly completeness. Briefly, completeness analysis is based on two types of non-redundant element sets, derived by merging overlapping genomic coordinates of transcripts or exons within each transcriptome assembly. Undirected graphs are independently constructed for transcript and exon sets, with edges indicating coordinate overlaps between sets across transcriptomes. In each graph, overlapping element sets are grouped into connected components, with the longest set designated as the representative. The completeness of each set is quantified as its length relative to the representative set in the corresponding group. Relative transcript and exon scores are computed as the mean completeness of the respective element sets, providing complementary insights into assembly quality. The transcript score captures large-scale structural issues, such as missing isoforms and fragmentation, while the exon score detects partial incompleteness and localized errors.

CATS-rb is primarily designed to estimate assembly completeness relative to the supplied transcriptome assemblies. However, the analysis can be supplemented using non-redundant reference element sets derived from genomic annotation. In this mode, assembly element set groups are defined according to shared overlaps with reference sets. Annotation-based element scores are calculated analogously to the relative scores, providing absolute completeness measures.

### CATS-rb performance assessment

**Fig. 6:**
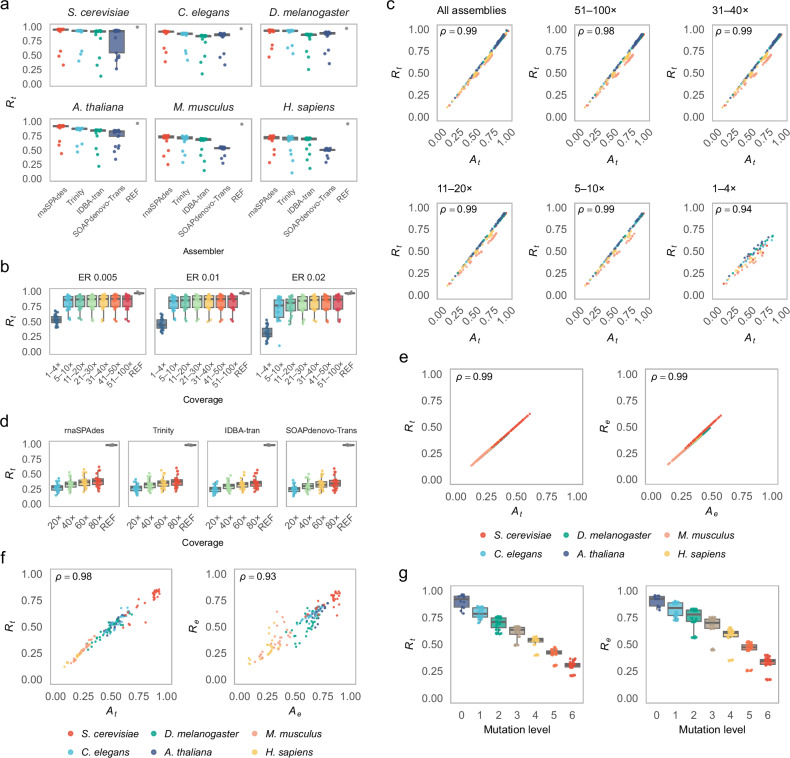
CATS-rb performance assessment. **a** Distribution of relative transcript scores across species and assemblers in controlled simulations (*n* = 21 assemblies per group). Colors indicate assemblers: red = rnaSPAdes, light blue = Trinity, green = IDBA-tran, dark blue = SOAPdenovo-Trans; gray indicates the reference transcriptome. **b** Distribution of relative transcript scores by coverage and error rate in controlled simulations (*n* = 24 assemblies per group, *n* = 6 reference transcriptomes). Colors denote coverage: dark blue = 1–4×, light blue = 5–10×, green = 11–20×, brown = 21–30×, yellow = 31–40×, orange = 41–50×, red = 51–100×; gray indicates the reference transcriptomes. **c** Correlation between relative and annotation-based transcript scores in assemblies with decreasing coverage (indicated in subplot titles). **d** Distribution of relative transcript scores across assemblers and coverage in realistically simulated libraries (*n* = 24 assemblies per group, *n* = 6 refeence transcriptomes). Colors represent coverage: light blue = 20×, green = 40×, yellow = 60×, red = 80×; gray indicates the reference transcriptomes. **e** Equivalent correlations to (**c**) in the realistically simulated dataset. **f** Equivalent correlations to (**c**) and (**e**) in the public dataset. In (**c**), (**e**), and (**f**), colors represent species: red = *Saccharomyces cerevisiae*, light blue = *Caenorhabditis elegans*, green = *Drosophila melanogaster*, dark blue = *Arabidopsis thaliana*, salmon = *Mus musculus*, yellow = *Homo sapiens*. **g** Distribution of relative scores in assemblies with multiplicative mutations. Mutation level 0 corresponds to *n* = 12 native assemblies, while levels 1–6 each encompass *n* = 36 assemblies. Colors represent mutation levels: dark blue = 0, light blue = 1, green = 2, brown = 3, yellow = 4, orange = 5, red = 6. Boxplots represent the median and IQR, with whiskers extending to ±1.5 × IQR. Points denote individual assemblies. *R*_*t*_ = relative transcript score, REF = reference transcriptome, ER = error rate, *A*_*t*_ = annotation-based transcript score, *R*_*e*_ = relative exon score*, A*_*e*_ = annotation-based exon score, *ρ* = Spearman correlation coefficient.

CATS-rb benchmark was performed on an extensive dataset of simulated and public RNA-seq libraries (Fig. 6), previously used in CATS-rf evaluation. We first examined the general features of CATS-rb metrics using the complete assembly set from controlled simulations. This dataset exhibited low intra-library coverage variability and included all reference transcripts, enabling a detailed assessment of CATS-rb scores across isolated effects of diverse assembly characteristics. Relative transcript and exon scores varied substantially across assemblers and species, with complex transcriptomes generally showing worse performance (Fig. 6a, Supplementary Fig. 4). Consistent with the reference-free pipeline, rnaSPAdes and Trinity outperformed IDBA-tran and SOAPdenovo-Trans. Reference assemblies yielded consistently high relative scores (transcript: 0.95–0.99, exon: 0.97–0.99). Assemblies generated at the lowest coverage levels showed the worst performance, with transcript and exon scores typically plateauing beyond 5–10× coverage (Fig. 6b, Supplementary Fig. 5). However, assemblies with the highest sequencing error rate continued to significantly improve with increasing depth.

CATS-rb relative scores were first evaluated across the complete controlled dataset and five assembly subsets with decreasing coverage for each species. The complete dataset included the reference transcriptome and all assemblies from controlled simulations. In contrast, each subset comprised only assemblies from controlled simulations and was defined by the coverage range of its highest-coverage assemblies, ranging from 51–100× to 1–4×. We observed a strong correlation between relative and annotation-based scores across the full dataset and all subsets (Fig. 6c, Supplementary Fig. 6). The stated association was strong even in the lowest-coverage subset (transcript: *ρ* = 0.94, 95%CI 0.90–0.96, exon: *ρ* = 0.95, 95%CI 0.93–0.97). These results indicate that relative CATS-rb scores remain stable across varying levels of assembly quality.

Relative CATS-rb scores were further examined using both realistically simulated and public datasets, each reflecting multifold coverage variability and a lower proportion of expressed transcripts. In the realistic simulations, CATS-rb was first applied to the complete per-species assembly sets, including all libraries and the appropriate reference transcriptome. These assemblies generally exhibited lower completeness scores than the assemblies from controlled simulations (Fig. 6d, Supplementary Fig. 7). Scores increased with greater sequencing depth and were higher for assemblies generated by rnaSPAdes and Trinity. CATS-rb was next applied to assemblies from individual realistically simulated libraries alongside the corresponding reference transcriptome. In this setting, assemblies exhibited a strong correlation between relative and annotation-based scores (transcript: *ρ* = 0.99, 95%CI 0.98–0.99, exon: *ρ* = 0.99, 95%CI 0.98–0.99, Fig. 6e). CATS-rb was also used to evaluate assemblies from each public library paired with the reference transcriptome. The public dataset displayed similarly strong correlations for transcript (*ρ* = 0.98, 95%CI 0.97–0.98) and exon (*ρ* = 0.93, 95%CI 0.91–0.95) scores (Fig. 6f).

To further evaluate its performance, CATS-rb was applied to assembly groups including native and mutated assemblies, the latter comprising six levels of multiplicative mutations. CATS-rb successfully captured the introduced errors, as reflected by a gradual decline in relative scores with increasing mutation levels (Fig. 6g).

**Fig. 7:**
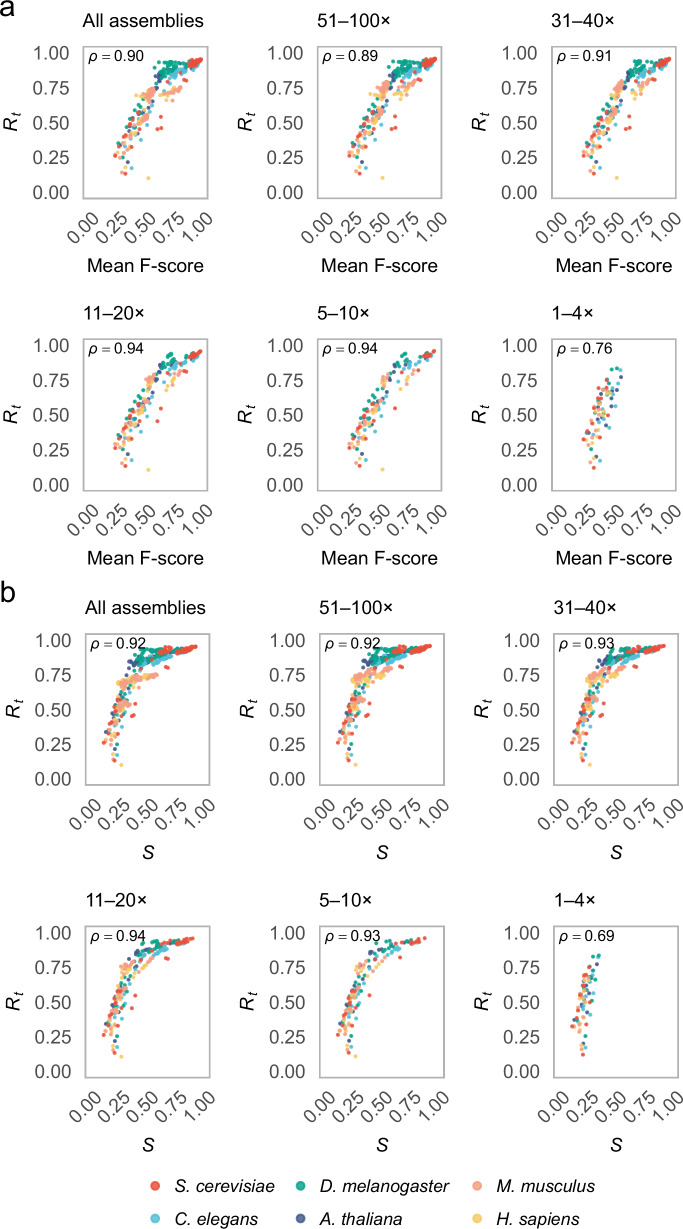
Association between transcriptome assembly completeness and the accuracy of assembled transcripts. **a** Correlation between relative CATS-rb transcript scores and mean transcript F-scores across assemblies with decreasing coverage (indicated in subplot titles). **b** Correlation between relative transcript scores and CATS-rf assembly scores across assemblies with decreasing coverage. Colors represent species: red = *Saccharomyces cerevisiae*, light blue = *Caenorhabditis elegans*, green = *Drosophila melanogaster*, dark blue = *Arabidopsis thaliana*, salmon = *Mus musculus*, yellow = *Homo sapiens. R*_*t*_ = relative transcript score, *S* = CATS-rf assembly score, *ρ* = Spearman correlation coefficient.

We also evaluated the consistency of assembly rankings by accuracy of assembled transcripts (mean F-scores, CATS-rf assembly scores) and assembly completeness (relative CATS-rb scores) in the controlled dataset (Fig. 7). Relative CATS-rb transcript scores exhibited a strong association with mean transcript F-scores across the full dataset (*ρ* = 0.90, 95%CI 0.88–0.91) and all coverage subsets (Fig. 7a). Analogous results were observed for relative exon scores (Supplementary Fig. 8). Even in the lowest-coverage subset, observed correlations remained strong for both transcript (*ρ* = 0.76, 95%CI 0.64–0.85) and exon (*ρ* = 0.79, 95%CI 0.68–0.86) scores. Furthermore, strong correlation between CATS-rb relative transcript scores and CATS-rf assembly scores was observed in the full dataset (*ρ* = 0.92, 95%CI 0.91–0.93) and in all assembly subsets, including the lowest-coverage subset (*ρ* = 0.69, 95%CI 0.54–0.79) (Fig. 7b). Similar trends were observed for exon scores (Supplementary Fig. 9). In the lowest-coverage subset, CATS-rb scores were notably higher than both the mean transcript F-scores and the CATS-rf assembly scores.

Lastly, we assessed the performance of CATS-rb in detecting chimerism by artificially merging transcripts from reference assemblies (Supplementary Fig. 10). CATS-rb demonstrated strong performance in classifying chimerism as a structural inconsistency, exhibiting high sensitivity (93.5%–96.8%) and specificity (97.0%–99.8%) across all assemblies.

## Discussion

This study presents CATS, a framework for transcriptome assembly evaluation combining reference-free (CATS-rf) and reference-based (CATS-rb) approaches. CATS-rf leverages short-read RNA-seq mappings to detect specific transcript errors through interpretable score components, thereby evaluating the quality of assembled transcripts. While validated using Illumina data, CATS-rf is compatible with all short-read sequencing platforms. In contrast, CATS-rb evaluates assemblies by aligning transcripts to a reference genome, providing robust completeness scores without relying on genomic annotation. CATS-rb does not require input RNA-seq reads, making it applicable to any assembly type or sequencing technology, including de novo, genome-guided, short-read, long-read, and hybrid approaches.

CATS-rf demonstrated superior transcript-level metrics compared to existing reference-free transcriptome assessment tools across a broad range of simulated libraries. Although the controlled simulations did not capture realistic coverage variability, they provided a framework for isolating the effects of diverse conditions on CATS scoring. When compared to existing transcriptome assessment tools, CATS-rf delivered significantly more reliable quality metrics at non-minimum coverage settings across all analyzed species and assemblers, while remaining highly competitive at the lowest depth (1-4×). The realistic simulations extended these findings to more biologically plausible datasets featuring high intra-library coverage variation. Under these conditions, CATS-rf maintained strong associations with transcript quality, surpassing RSEM-EVAL and TransRate across the vast majority of the analyzed species, sequencing depths, and assemblers.

While CATS-rf continued to significantly outperform RSEM-EVAL and TransRate on public datasets, its quality estimates exhibited weaker correlations with assembly accuracy. Benchmarking on real datasets remains intrinsically difficult due to the absence of a complete ground-truth transcriptome, as transcripts absent from the reference are assigned inaccurate F-scores. To mitigate this, we restricted the benchmark to transcripts represented in the reference transcriptome, as determined by alignment-based criteria. However, this approach may have excluded poorly assembled transcripts that failed to align to the reference transcriptome. This likely biased the evaluation, as the benchmarking set was enriched for higher-quality transcripts. As such, these results should be interpreted in hand with the findings from simulated assemblies.

Reference-free approaches are generally limited in their ability to estimate absolute transcript quality across different libraries^13,14^. This is particularly evident for RSEM-EVAL, whose transcript-level metrics primarily reflect each transcript’s contribution to the overall assembly score rather than its intrinsic accuracy. On the other hand, CATS-rf and TransRate were designed to directly estimate transcript-level quality by examining read mapping evidence. Accordingly, CATS-rf was able to consistently rank assemblies according to their objective transcript accuracy across diverse conditions. Moreover, CATS-rf outperformed transcript scores provided by both RSEM-EVAL and TransRate. This suggests that transcript error estimates based on read mapping evidence implemented in CATS-rf can provide a reliable proxy for absolute accuracy of assembled transcripts.

Although CATS-rf and TransRate are conceptually similar in their use of read mapping and transcript-level scoring to quantify assembly errors, they differ substantially in underlying methodologies. The primary distinctions involve approaches to read mapping, handling of multimapped reads, definition of transcript score components, and calculation of the overall assembly score (Supplementary Table 1). CATS-rf demonstrated better performance in detecting assembly errors, as reflected by stronger correlations between its quality metrics and transcript accuracy under controlled and realistic settings. The improvement stems from the design of CATS-rf score components, which effectively separate mapping inconsistencies due to sequencing noise and transcript variation from those caused by misassembly. Accordingly, CATS-rf score components showed high sensitivity to their targeted error types in transcripts with sufficient coverage. However, this sensitivity is constrained at minimum depth, as limited read evidence can obscure the patterns required to identify specific misassembly events. In our simulation experiments, this limitation likely led to slightly lower accuracy in transcripts with low coverage.

Regarding specificity of CATS-rf score components, read mapping evidence associated with each component cannot be solely assigned to a single misassembly event, as multiple error types can contribute to the observed inconsistencies. However, low intercorrelations between the score components across diverse assembly conditions, along with distinct clustering of error types in PCA, suggest that different error classes yield characteristic score component distributions.

While CATS-rf score components penalize unsupported fusion sites, CATS-rf does not directly detect chimerism. This limitation is likely minor, as studies report a low incidence of chimeric artifacts produced by modern assemblers^10,12,18^. In contrast, TransRate computes a chimerism score based on the uniform coverage assumption^14^, which is unrealistic due to fragmentation and GC content biases^19–21^. Meanwhile, the reference-based approach employed in CATS-rb demonstrated strong performance in identifying chimerism as a form of structural inconsistency. However, the obtained metrics should be interpreted in the context of rare-event classification.

CATS-rb directly estimates transcriptome assembly completeness without relying on reference transcriptomes or genomic annotation, making it suitable for the analysis of poorly annotated organisms and non-coding transcripts. Evaluation on controlled and realistic datasets revealed a strong correlation between CATS-rb relative and annotation-based scores. CATS-rb also showed high sensitivity to assembly errors, as reflected by significantly reduced scores in mutated assemblies. Furthermore, reference transcriptomes consistently received near-maximum scores, suggesting minimal score inflation. These findings indicate that the tool effectively estimates transcript completeness on complex and low-quality datasets. As such, we propose that CATS-rb is valuable in three scenarios: evaluating assemblies generated from the same library, comparing transcriptomic content across libraries, and assessing absolute completeness using a reference transcriptome or genomic annotation.

Additionally, CATS-rb scores were closely associated with F-scores and CATS-rf assembly scores. While this association remained strong across all coverage levels, the absolute values of CATS-rb scores significantly differed from F-scores and CATS-rf scores at the lowest coverage. This discrepancy likely reflects the differing evaluation scope: both CATS-rf and F-scores assess transcript-level accuracy, whereas CATS-rb primarily captures assembly completeness. Despite assessing distinct aspects of assembly quality, CATS-rf and CATS-rb produced highly consistent rankings of the analyzed assemblies. However, joint interpretation of CATS-rf and CATS-rb scores offers a complete representation of assembly quality.

The performance of both tools within the CATS framework is intrinsically tied to their respective alignment strategies. CATS-rf utilizes Bowtie2 with optimized parameters that balance between error detection and mapping stringency. In parallel, CATS-rb leverages Spaln for robust transcript-to-genome alignment, incorporating species-specific parameters that can be user- adjusted. While we do not claim these aligners are universally optimal, both are regarded as highly accurate^22,23^, and the selected parameters have been optimized for transcriptome assembly evaluation.

Benchmarking both tools revealed substantial variation in assembly quality across species, with complex transcriptomes consistently yielding lower scores. This trend reflects the assembly challenges posed by isoform diversity, paralogous gene families, and repetitive sequences^1,7,9^. Both tools ranked rnaSPAdes and Trinity above IDBA-tran and SOAPdenovo-Trans, agreeing with recent benchmarking studies^7,9,11,12^. However, this result should be validated using assemblies generated with a broader range of k-mer lengths. In controlled simulations, increasing sequencing depth beyond a certain threshold did not significantly improve assembly scores, particularly in low-error datasets. Similarly, coverage increases in realistic simulations yielded progressively smaller gains in assembly quality, as most reads originated from strongly expressed transcripts. These results are consistent with studies highlighting the diminishing returns of deeper sequencing for accurate transcript reconstruction^19,24^. Our benchmarks also underscore the substantial impact of coverage variability on assembly quality. Realistic datasets with multifold coverage variation displayed lower accuracy compared to controlled datasets with non-minimal coverage. Nevertheless, even the idealized assemblies from controlled simulations exhibited variable accuracy and did not achieve the completeness observed in reference assemblies.

In summary, CATS provides a comprehensive framework for evaluating transcriptome assemblies, combining the strengths of reference-free and reference-based approaches. Its consistent performance across diverse conditions makes it well-suited for a broad range of transcriptomic studies. By offering interpretable and robust metrics, CATS facilitates reliable quality control of transcriptome assemblies.

## Methods

### CATS-rf algorithm overview

CATS-rf generates transcript-level quality metrics using short-read RNA-seq data employed in the transcriptome assembly process. The pipeline begins by mapping the reads to the evaluated assembly using Bowtie2^22^, with parameters optimized for accurate detection of assembly errors. Read alignment is performed in global mode using the sensitive preset. To accommodate transcripts with multiple isoforms, the default maximum number of alignments per read is set to 10. A mismatch penalty of 2 is applied to allow the detection of potentially inaccurate transcript regions, while controlling for false-positive alignments. Secondary alignments with edit distance exceeding a defined threshold are discarded, reducing off-target mappings. This threshold is set to the sum of the maximum edit distance of the read and 10% of mean read length.

Reads that map to multiple transcripts are probabilistically assigned to a single transcript based on expression levels calculated by Kallisto using default parameters^25^. Specifically, the probability *P* of assigning a read to transcript *t* is proportional to transcript abundance, measured in transcripts per million (TPM):

1 $$P=\,\frac{\sqrt{{{{\rm{TP}}}}{{{{\rm{M}}}}}_{{{{\rm{t}}}}}}}{{\sum }_{{t}^{{\prime} }{{{\rm{\epsilon }}}}\,S}\sqrt{{{{\rm{TP}}}}{{{{\rm{M}}}}}_{{{{{\rm{t}}}}}^{{\prime} }}}}$$

where *S* is the set of all candidate transcripts *t’* for a given read.

The introduced transformation of TPM aims to increase the effective number of assigned reads in lowly-expressed transcripts. To ensure mapping consistency, CATS-rf enforces co-assignment of paired reads. If read pairs map to different transcripts, both are reassigned to the most abundant shared transcript. This eliminates artifacts that could bias downstream scoring components based on pair mapping.

CATS-rf computes transcript-level quality scores by analyzing per-base coverage patterns and the consistency of paired-read mappings. Per-base transcript statistics are extracted from read mappings using pysamstats^26^. Four score components are defined, each corresponding to distinct types of assembly errors. Considering that CATS-rf score components include several tunable parameters, detailed descriptions of their default values and the underlying rationale are provided in Supplementary Table 2. It should be noted that none of these parameters were specifically optimized for the assemblies evaluated in this study. To demonstrate parameter robustness, we performed a random grid-search analysis evaluating the impact of parameter variation on the accuracy of each score component (Supplementary Fig. 11, Supplementary Methods).

The coverage score component *S*_*c*_ penalizes transcript regions with low read coverage, primarily aiming to capture unsupported insertions and redundancy. Low coverage regions (LCR) are identified by calculating the mean per-base coverage within rolling windows of a fixed length and merging all contiguous bases whose corresponding window-mean coverage falls below a specified threshold. Each LCR is assigned a coverage penalty *P*_*c*_ calculated as:

2 $$P_c={\sum }_{n=1}^{{L}_{c}}f\left({C}_{n}\right)+{E}_{c} * {L}_{c}$$

Here, *C*_*n*_ denotes the coverage of the n_th_ base. *E*_*c*_ represents the LCR extension penalty, *L*_*c*_ corresponds to LCR length, and *f*(*C*_*n*_) is a function of per-base coverage defined as:

3 $$f({C}_{n})=\left\{\begin{array}{c}1,\,\,\,\,{{{\rm{if}}}}\,{C}_{n}=0\\ \frac{1}{W * \,{C}_{n}},\,\,{{{\rm{if}}}}\,{C}_{n} > 0\end{array}\,\right\}\,$$

where *W* is the base coverage weight, which scales the penalty applied to bases with non-zero coverage.

The coverage penalty incorporates per-base coverage information (*f*(*C*_*n*_)) and an extension component (*E*_*c*_*)*, reflecting both the severity and extent of reduced coverage within the LCR. This formulation of LCRs and the associated coverage penalty is designed to detect extended unsupported regions indicative of assembly errors. In contrast, read- and allele-specific indels incur minimal penalties, which is appropriate as such events reflect false-positive coverage drops rather than misassembly.

The coverage component is defined as the complement of the normalized sum of coverage penalties:

4 $${S}_{c}=1-\sqrt{\frac{{\sum }_{n=1}^{{N}_{c}}{P}_{c,n}}{{L}_{t} * ({E}_{c}+1)}}$$

where *N*_*c*_ is the number of LCRs per transcript and *L*_*t*_ denotes transcript length.

The coverage component normalizes the total coverage penalty per transcript such that a value of zero corresponds to completely uncovered transcripts, while a value of one is assigned to transcripts without LCRs.

The accuracy score component *S*_*a*_ identifies regions exhibiting inaccurate transcript sequence. Accuracy is calculated as the fraction of aligned read bases matching the transcript base. Low-accuracy regions (LAR) are defined by computing the mean accuracy of covered bases within rolling windows of a fixed length and merging all contiguous bases with the corresponding window-mean accuracy below a specified threshold. The definition is refined by the exclusion of fully accurate bases on LAR ends. Each LAR is assigned an accuracy penalty *P*_*a*_ given by:

5 $$P_a=\mathop{\sum }_{n=1}^{{L}_{a}}(1-{A}_{n})+{E}_{a} * {L}_{a}$$

where *A*_*n*_ represents the accuracy of the n_th_ base, *E*_*a*_ denotes the LAR extension penalty, and *L*_*a*_ corresponds to LAR length.

Analogous to its coverage counterpart, the accuracy penalty integrates per-base accuracy with an extension component to capture both the degree and scale of reduced accuracy. This formulation penalizes extended inaccurate regions, indicative of misassembly events such as the collapse of similar isoforms or repetitive sequences. In contrast, isolated base-level discrepancies, including sequencing errors or single nucleotide polymorphisms, are minimally penalized as they do not reflect assembly errors.

The accuracy component is defined as the complement of the normalized sum of accuracy penalties:

6 $${S}_{a}=1-\sqrt{\frac{{\sum }_{n=1}^{{N}_{a}}{P}_{a,n}}{{L}_{{{\mathrm{cov}}}} * ({E}_{a}+1)}}$$

where *N*_*a*_ is the number of LARs per transcript and *L*_cov_ denotes the number of covered transcript bases.

The accuracy component normalizes the total accuracy penalty by the number of covered bases per transcript, assigning a value of zero to the theoretical scenario in which all covered bases exhibit zero accuracy, and a value of one to transcripts without LARs.

If a paired-end library is provided, CATS-rf calculates the local fidelity and integrity score components based on paired-read mapping patterns. The local fidelity score component *S*_*l*_ detects mapping inconsistencies of read pairs within individual transcripts, capturing structural errors such as deletions, inversions, or translocations. This component incorporates three transcript-level metrics: number of reads whose pair fails to map to the assembly (*N*_*u*_), number of pairs mapping to the same transcript in unexpected orientations (*N*_*o*_), and total distance penalty (*P*_*d*_) per transcript. Unexpected orientations are identified based on library strandness, which can be supplied by the user or automatically detected.

The distance penalty *P*_*d*_ is computed for each properly oriented pair mapped to the same transcript using a segmented function of the observed pair distance *d*:

7 $${P}_{d}=\left\{\begin{array}{c}0,\quad{{{\rm{ if }}}}\;d < {D}_{1}\\ \frac{d}{D_2},\;{{{\rm{if }}}}\;{D}_{1}\le d\le {D}_{2}\\ 1,\;\;{{{\rm{if}}}}\;d > {D}_{2}\end{array}\right\}$$

where *D*_1_ and *D*_2_ denote distance outlier thresholds are defined as:

8 $${D}_{1}={{{{\rm{Q}}}}}_{3}\left(d\right)+\,{M}_{1} * ({{{\rm{IQR}}}}\left(d\right)+C)$$

9 $${D}_{2}={{{{\rm{Q}}}}}_{3}\left(d\right)+\,{M}_{2} * ({{{\rm{IQR}}}}\left(d\right)+C)$$

Here, Q_3_(*d*) is the third quartile and IQR(*d*) is the interquartile range of the observed pair distance. *M*_1_ and *M*_2_ multiplicative factors reduce the impact of false-positive outliers, which may result from variations in library preparation. The correction factor *C* ensures threshold robustness in libraries with a high proportion of overlapping read pairs. This definition penalizes pairs exhibiting distance outliers relative to the expected interpair distance distribution, where the outlier threshold is determined by *D*₁, and the penalty scailing is controlled by *D*₂. Distance penalty is by definition normalized to the interval [0, 1],

The stated mapping inconsistencies are combined into the local fidelity component:

10 $${S}_{l}=1-\,\sqrt{\frac{{N}_{u}+2{N}_{o}+2 \mathop{\sum }_{n=1}^{{N}_{p}}{P}_{d,n}}{{N}_{r}}}$$

where *N*_*p*_ refers to the number of properly oriented pairs mapped to the same transcript and *N*_*r*_ is the total number of mapped reads per transcript. The local fidelity component quantifies pair mapping consistency by normalizing the number of improperly mapped reads within a transcript against the total number of mapped reads per transcript to the interval [0, 1].

The integrity score component *S*_*i*_ captures transcript fragmentation by detecting bridging events, defined as read pairs mapped to separate transcripts. For each bridging event, a bridge penalty *P*_*b*_ is computed as the mean of the relative distances of each read from the center of its respective transcript. The distances are normalized such that a value of 0 corresponds to the transcript center and a value of 1 to the transcript ends. This formulation assumes that bridging read pairs located near transcript ends are more indicative of genuine fragmentation. Bridge index *B* for a transcript is calculated by normalizing the sum of bridge penalties against the total number of mapped reads per transcript:

11 $$B=1-\,\frac{1}{{N}_{r}}\mathop{\sum }_{n=1}^{{n}_{b}}{P}_{b,n}$$

where *n*_*b*_ is the number of bridging events per transcript.

The integrity component is defined by a sigmoid transformation of the bridge index:

12 $${S}_{l}=\,\frac{{B}^{\alpha }}{{B}^{\alpha }+{(1-\,B)}^{\beta }}$$

Here, *α* and *β* denote tunable compression factors that regulate sensitivity to fragmentation patterns. The transformation compensates for consistent mapping of read pairs to accurately assembled interior transcript regions, inherently weakening the fragmentation signal.

The transcript score *S*_*t*_ is calculated as the product of the defined score components:

13 $${S}_{t}={S}_{c} * {S}_{a} * {S}_{l} * {S}_{i}$$

The assembly score *S* is calculated as the mean of individual transcript scores:

14 $$S=\frac{1}{{N}_{t}}\mathop{\sum }_{n=1}^{{N}_{t}}{S}_{t,n}$$

where *N*_*t*_ is the total number of transcripts. The assembly score estimates the quality of the assembled transcripts, equally weighting distinct assembly error types. By definition, transcript and assembly scores are normalized to the interval [0, 1], where higher values correspond to greater quality. Completely uncovered transcripts are assigned a transcript score of zero, as their coverage component is set to zero, with other components left undefined.

Because the assembly score reflects only the accuracy of the assembled transcripts, CATS-rf further provides an estimate of assembly completeness relative to the input reads as a separate metric. This completeness estimate is expressed as the proportion of reads that map to the assembled transcripts.

Additionally, CATS-rf computes and visualizes a comprehensive set of mapping and positional metrics to support quality assessment. These include coverage and accuracy statistics such as the number of uncovered and inaccurate bases, mean transcript coverage and accuracy, per-base and positional distributions of coverage and accuracy, as well as length distributions of LCRs and LARs. Paired-read mapping metrics encompass the number of inconsistently mapped read pairs, bridging events, and potentially fragmented transcripts.

### CATS-rb algorithm overview

CATS-rb performs reference-based transcriptome assembly assessment by aligning transcripts to a reference genome using Spaln, a highly accurate spliced aligner^23^. The pipeline comprises three core tools that perform genome index generation, transcriptome mapping, and comparative mapping analysis. During index generation, users can define the maximum gene size. Mapping parameters are highly customizable to accommodate assembly-specific features, including library strandness, species-specific presets, maximum number of alignments per transcript, minimum intron size, splice site recognition criteria, and weighting of alignment scores based on coding potential and translation signals.

CATS-rb workflow evaluates assemblies in terms of accuracy and completeness. The analysis begins by processing exon-level alignment output from Spaln, which is used to reconstruct transcript-level mappings. Exons originating from the same transcript are assigned to separate transcript mappings under the following conditions: 1) the exon overlaps the preceding exon in transcript coordinates by more than 50% of the preceding exon’s length; 2) the exon maps to a different genomic scaffold or strand than the preceding exon; or 3) the exon maps to the same scaffold and strand, but the distance to the preceding exon exceeds the intron length threshold.

The pipeline evaluates multiple aspects of assembly mapping, including transcript mappability, multimapping, exon count per transcript, exon length distribution, isoform representation, and structural consistency. A transcript is classified as structurally inconsistent if its alignment proportion falls below a predefined threshold or if it contains regions that map to disjunct genomic loci. The latter is assessed by identifying transcript segments that overlap by less than a specified proportion of their lengths and map either to different scaffolds, to opposite strands on the same scaffold, or to distant regions on the same scaffold and strand (beyond the intron length threshold). Flagged pairs are excluded if one segment is shorter than 10% of the other segment’s length. This definition is further refined by removing cases where a separately mapped transcript segment covers more than 90% of each segment in the marked pair (in transcript coordinates). The described structural consistency criteria are primarily designed to detect chimerism, while also capturing events resulting in significant mismatches to the reference genome, arising either from assembly errors or genuine biological variation.

The primary contribution of CATS-rb is the analysis of assembly completeness, which introduces two types of element sets as units for transcriptome assembly comparison. Precisely, transcript and exon sets are defined by merging the overlapping transcript or exon genomic coordinates within a given transcriptome into non-redundant segments. For multimapped transcripts, element sets are constructed using the most accurate mapping, with ties resolved by selecting the matching mapping across the analyzed transcriptomes. CATS-rb builds separate undirected graphs for both element set types, in which vertices represent element sets and edges indicate overlaps between sets of the compared assemblies. Overlaps are determined using a predefined overlap length threshold for edge specification. Element set groups are defined as connected graph components. If multiple sets from the same assembly fall within a single group, only the longest is retained. The group representative is defined as the longest set in the group. Element set completeness *c* is calculated as the length of the set relative to the representative set of its group. Sets absent from a group are assigned a completeness of 0. The relative element score *R* for each assembly is computed as the mean element set completeness across all groups:

15 $$R=\,\frac{1}{{N}_{g}}{\sum }_{n=1}^{{N}_{g}}{c}_{n}$$

Here, *N*_*g*_ denotes the number of element set groups. This procedure is applied independently to transcript and exon sets to produce relative transcript and exon scores.

The completeness assessment also provides a detailed evaluation of element set distribution across the compared transcriptome assemblies. This includes an in-depth analysis of missing, common, and unique element sets, along with element set UpSet plots and Venn diagrams. Assemblies are also hierarchically clustered based on element set completeness using complete linkage clustering with the Euclidean distance metric.

The presented method evaluates assembly completeness relative to the compared transcriptomes without requiring external genomic annotation. Additionally, CATS-rb can perform an annotation-based analysis using non-redundant reference element sets. These sets are constructed by separately merging overlapping transcript coordinates and overlapping exon coordinates defined by external genomic annotation. The annotation-based workflow follows the same principles as relative completeness evaluation, while grouping transcriptome assembly element sets based on shared overlaps with reference sets. As such, reference sets are considered the representative for each assembly set group. Annotation-based element scores *A* are calculated analogously to relative element scores, providing an absolute metric of assembly completeness.

Genomic annotation is also used to calculate element set sensitivity and specificity for each transcriptome assembly. Sensitivity is defined as the proportion of reference sets matched by assembly element sets, while specificity reflects the proportion of assembly element sets matched by reference sets. A set is considered matched if its genomic coordinates cover more than a predefined fraction of the corresponding set’s length.

### RNA-seq library preparation

We performed an extensive evaluation of CATS performance using a diverse set of RNA-seq libraries simulated with the Polyester R package (R version 4.4.2, package version 1.32.0)^27,28^. First, we designed an idealized simulation framework that orthogonally varied transcriptome complexity, narrow coverage ranges, sequencing error rate, and assembler choice. A total of 126 paired-end, unstranded libraries were simulated from coding and non-coding transcripts of six species*: Saccharomyces cerevisiae, Caenorhabditis elegans*, *Drosophila melanogaster*, *Arabidopsis thaliana*, *Mus musculus*, and *Homo sapiens* (Supplementary Table 3). For each species, 21 libraries were generated by combining seven transcript coverage levels with three sequencing error rates. Coverage levels were simulated by randomly sampling baseline transcript coverages from the following ranges: 1–4×, 5–10×, 11–20×, 21–30×, 31–40×, 41–50×, and 51–100×. All reference transcripts were treated as expressed. A uniform error distribution model was applied, with error rates set to 0.005, 0.01, or 0.02 for each coverage level.

We also benchmarked CATS on realistically simulated libraries exhibiting multifold variation in sequencing coverage. A total of 96 libraries were generated, spanning six species and four coverage levels, each represented by four replicates (Supplementary Table 4, Supplementary Table 5). A uniform base-level error rate of 0.005 was applied. Sequencing depth was determined by a target mean coverage level *C* chosen from four possible values: 20×, 40×, 60×, and 80×. The number of read pairs per library *N*_*r*_ was calculated as

16 $${N}_{r}=\,\frac{C * \mathop{\sum }_{n=1}^{{n}_{e}}{L}_{t,n}}{2 * {L}_{r}}$$

where *n*_*e*_ denotes the number of expressed transcripts, *L*_*t*,*n*_ the length of the nth transcript, and *L*_*r*_ the read length.

To reflect biological variability, the proportion of expressed transcripts per replicate was randomly sampled within species-specific ranges: *S. cerevisiae*: 70–90%, *C. elegans*, *D. melanogaster*, and *A. thaliana*: 30–50%, *M. musculus* and *H. sapiens*: 20–40%. For each expressed transcript, relative abundance values were drawn from a log-normal distribution with parameters meanlog = 1 and sdlog = 2, corresponding to a 10^4^-10⁵-fold range in coverage within a single library (Supplementary Fig. 12). Abundance values were subsequently scaled by transcript length. The number of reads assigned to each transcript was determined by multiplying relative abundance values by total library size. In both controlled and realistic simulations, reads were 100 bp in length, and fragments were generated using an RNA fragmentation protocol based on the empirical fragment length distribution provided by Polyester.

In addition to the simulated dataset, we benchmarked CATS on 42 publicly available RNA-seq libraries from the same six species (Supplementary Table 6). These libraries were obtained from the Sequence Read Archive (SRA) database using the SRA Toolkit (version 3.2.0)^29^. Adapter sequences were removed and low-quality bases trimmed from all public libraries using Trim Galore (version 0.6.1) alongside cutadapt (version 4.6) with the parameters -stringency 5 -q 5^30^. Transcriptomes from both simulated and public libraries were assembled using four de novo assemblers: rnaSPAdes (version 3.15.4), Trinity (version 2.15.1), IDBA-tran (version 1.1.1), and SOAPdenovo-Trans (version 1.0.4), each with default parameters. This resulted in a total of 504 assemblies from controlled simulations, 384 assemblies from realistic simulations, and 168 assemblies generated from public libraries.

We also tested the ability of CATS to detect assembly errors. Firstly, we independently introduced varying levels of mutations into a subset of simulated assemblies, including insertions, mismatches, deletions, redundancy, and fragmentation. Mutations were simulated in 12 assemblies generated using rnaSPAdes and Trinity, derived from libraries representing the six earlier stated species, each with transcript coverage range of 21–30× and a sequencing error rate of 0.005. For every mutation type, four levels of severity were simulated. In the case of insertions, mismatches, and deletions, a single mutated region was introduced at a random position within the selected transcripts. The proportion of affected transcripts was set to 15%, 30%, 45%, and 60%, with the length of the mutated regions scaled accordingly (15%, 30%, 45%, or 60% of the transcript length). For mismatches, each base within the mutated region was assigned a 25% probability of substitution, with equal likelihood for each alternative base. Redundancy was simulated by duplicating a corresponding transcript subsequence beginning from the 5’ transcript end, with 20%, 40%, 60%, and 80% of transcripts containing duplicated regions covering 20–39%, 40–59%, 60–79%, and 80–99% of the transcript length, respectively. Fragmentation was simulated by splitting transcripts at a random position between the 40 and 60th percentiles of transcript length, affecting 20%, 40%, 60%, and 80% of transcripts, respectively.

In addition to isolated mutation types, we performed a multiplicative error simulation, applying all five mutation types sequentially to the same 12 assemblies. The mutations were applied in the following order: deletion, mismatch, insertion, fragmentation, and redundancy. The simulation was conducted at six mutation severity levels (10, 20, 30, 40, 50, and 60%), which controlled both the proportion of affected transcripts and the length of the affected region. To introduce stochastic variation, the multiplicative simulation was applied three times to each assembly. All mutation simulations were performed using custom R scripts and R package Biostrings (version 2.27.1)^31^.

### Performance evaluation of CATS-rf

To compare the performances of CATS-rf, RSEM-EVAL (version 1.11) and TransRate (version 1.0.4), we ran each tool on all simulated and public assemblies using their default parameters. For simulated assemblies, the fragment length distribution mean required by RSEM-EVAL was set to 180 bp, in accordance with the empirical distribution provided by Polyester. For public assemblies, fragment length distribution parameters were estimated using Kallisto (version 0.50.1). TransRate was obtained from a Github repository that addressed inaccurate results produced by the current official release^32^. To evaluate transcript-level accuracy, we used CRB-BLAST (version 1.0.0) to identify reciprocal best alignments between each tested and reference transcriptome^33^. For each aligned transcript pair, we calculated the F-score as the harmonic mean of precision and recall. Precision was calculated as the number of aligned bases relative to the tested transcript length, while recall was defined as the number of aligned bases relative to the reference transcript length. Tested transcripts without hits were assigned an F-score of 0. For assemblies generated from realistically simulated and public libraries, CRB-BLAST was applied exclusively to transcripts represented in the reference transcriptomes. In simulated libraries, these transcripts were defined for each replicate by the simulation design. In public libraries, the represented transcripts were identified by alignment to the reference transcriptomes using BLAT (version 2.17.0) with sensitive parameters (-tileSize=8 -maxIntron=0)^34^, retaining only alignments with F-score greater than 0.5.

The performance of CATS-rf, RSEM-EVAL, and TransRate was evaluated with the Spearman correlation coefficient between transcript quality scores and F-scores. Transcript-level performance was assessed by calculating the stated correlations within each assembly. Assembly-level performance focused exclusively on the accuracy of assembled transcripts and was evaluated by correlating the mean transcript quality score of each assembly with its mean transcript F-score. Assembly-level correlations were compared using the two-tailed Meng’s z-test for overlapping correlations (R package cocor, version 1.1.4)^35^, with confidence intervals for Spearman’s rho calculated using Fisher’s z-transformation (R package DescTools, version 0.99.60)^36^. Additionally, we assessed the ability of CATS-rf to capture common assembly errors by comparing CATS-rf assembly scores and transcript-level score components across assemblies with increasing levels of simulated mutations. CATS-rf performance assessment was performed using custom R scripts with the following packages used for data manipulation and visualization: data.table (version 1.16.4)^37^, ggplot2 (version 3.5.1)^38^, ggcorrplot (version 0.1.4.1)^39^, cowplot (version 1.1.3)^40^, and ggbiplot (version 0.6.2)^41^.

### Performance evaluation of CATS-rb

The performance of CATS-rb was also evaluated using both simulated transcriptome assemblies and assemblies generated from public RNA-seq libraries. Reference genomes and annotations were obtained from Ensembl (Supplementary Table 7). Genome indexing, transcriptome mapping, and mapping comparison were performed using default CATS-rb parameters, with adjustments to maximum gene sizes and species-specific parameters (Supplementary Table 8). CATS-rb was first applied to the complete assembly set of each species from (i) controlled and (ii) realistic simulations, each paired with reference transcriptomes. Relative transcript and exon scores were analyzed for their association with library characteristics.

We further examined the robustness of relative transcript and exon scores by running CATS-rb on five assembly subsets. The subsets excluded reference transcriptomes and consisted of assemblies from controlled simulations with progressively lower transcript coverage. Each subset was defined by the coverage range of its highest-coverage assemblies: 51–100×, 31–40×, 11–20×, 5–10×, and 1–4×. Robustness of relative scores was assessed by examining their correlation with annotation-based scores. Relative and annotation-based score correlations were also evaluated on realistically simulated and public datasets, where CATS-rb was run separately for each library with the appropriate reference transcriptome.

We also assessed the sensitivity of CATS-rb to assembly errors by testing it on assemblies subjected to multiplicative mutations. Each tested set included the native assembly and triplicates of assemblies generated with six increasing levels of mutation intensity, previously used in CATS-rf evaluation.

To evaluate the ability of CATS-rb to classify chimerism as a structural inconsistency, we performed chimerism simulation by randomly merging transcript pairs from the reference transcriptomes of the six analyzed taxa into single transcripts. Prior to merging, random deletions were introduced at fusion sites of both transcripts, affecting up to 3% of each transcript length. The resulting assemblies contained 10% chimeric transcripts. CATS-rb performance evaluation was conducted using custom R scripts with the following packages used for data manipulation and visualization: data.table (version 1.16.4), ggplot2 (version 3.5.1), and cowplot (version 1.1.3).

### Reporting summary

Further information on research design is available in the Nature Portfolio Reporting Summary linked to this article.

## Supplementary information

Supplementary Information

Reporting Summary

Transparent Peer Review file

## Data Availability

All datasets analyzed in this study are publicly available. Reference transcriptome assemblies, genome sequences, and gene annotations used in RNA-seq library simulation and CATS benchmarking were obtained from Ensembl. Reference data for *S. cerevisiae* (R64-1-1), *C. elegans* (WBcel235), *D. melanogaster* (BDGP6.46), *M. musculus* (GRCm39), and *H. sapiens* (GRCh38) were downloaded from Ensembl release 111. Reference data for *A. thaliana* (TAIR10) were obtained from Ensembl Plants release 56. Public RNA-seq libraries used in CATS benchmarking are available via SRA under the following accession numbers: SRR32108057, SRR30685385, SRR27822254, SRR26147123, SRR26001199, SRR24356101, SRR10815431 (*S. cerevisiae*); SRR32732688, SRR32058534, SRR31107386, SRR12868377, SRR17736005, SRR5224028, SRR6266308 (*C. elegans*); SRR31530981, SRR29606991, SRR24876155, SRR23933588, SRR23870057, SRR20326862, SRR14560308 (*D. melanogaster*); SRR33339778, SRR30855151, SRR14160649, SRR24948904, SRR24636232, SRR13159213, SRR8422221 (*A. thaliana*); SRR32903297, SRR31588239, SRR27369179, SRR28908270, SRR18855849, SRR24134730, SRR13981556 (*M. musculus*); and SRR32139880, SRR31785722, SRR25646397, SRR25732618, SRR22548603, SRR8357441, SRR7741229 (*H. sapiens*). Complete CATS benchmark results generated in this study, including processed data and values underlying figures, have been deposited in Zenodo at 10.5281/zenodo.16837970^42^.

## Code availability

The CATS framework is implemented in R and Bash. The source code is available in the following repositories: CATS-rf: https://github.com/bodulic/CATS-rf (GitHub) and 10.5281/zenodo.16786177 (Zenodo)^43^. CATS-rb: https://github.com/bodulic/CATS-rb (GitHub) and 10.5281/zenodo.16786251 (Zenodo)^44^. Both tools are distributed under the MIT license. In addition, the code used to perform the CATS benchmark is available at: https://github.com/bodulic/CATS_benchmark (GitHub) and 10.5281/zenodo.16786150 (Zenodo)^45^.
