# Supplementary material for: Comprehensive assessment of transcriptome assembly quality using CATS
Source: Nat Commun. 2026 Apr 20;17:5419. doi: 10.1038/s41467-026-72171-8 (PMC13280353; doi:10.1038/s41467-026-72171-8)
Supplement: Supplementary file 1 — Supplementary Information [file 41467_2026_72171_MOESM1_ESM.pdf]

Comprehensive Assessment of Transcriptome Assembly Quality using CATS

Kristian Bodulić[1,2], Kristian Vlahoviček[2,*]

[1]Department for Bioinformatics and Statistics, University Hospital for Infectious Diseases "Dr. Fran Mihaljević", Mirogojska 8, 10000 Zagreb, Croatia

[2]Bioinformatics Group, Division of Molecular Biology, Department of Biology, Faculty of Science, University of Zagreb, 10000 Zagreb, Croatia

*Correspondence: kristian@bioinfo.hr

Supplementary Information

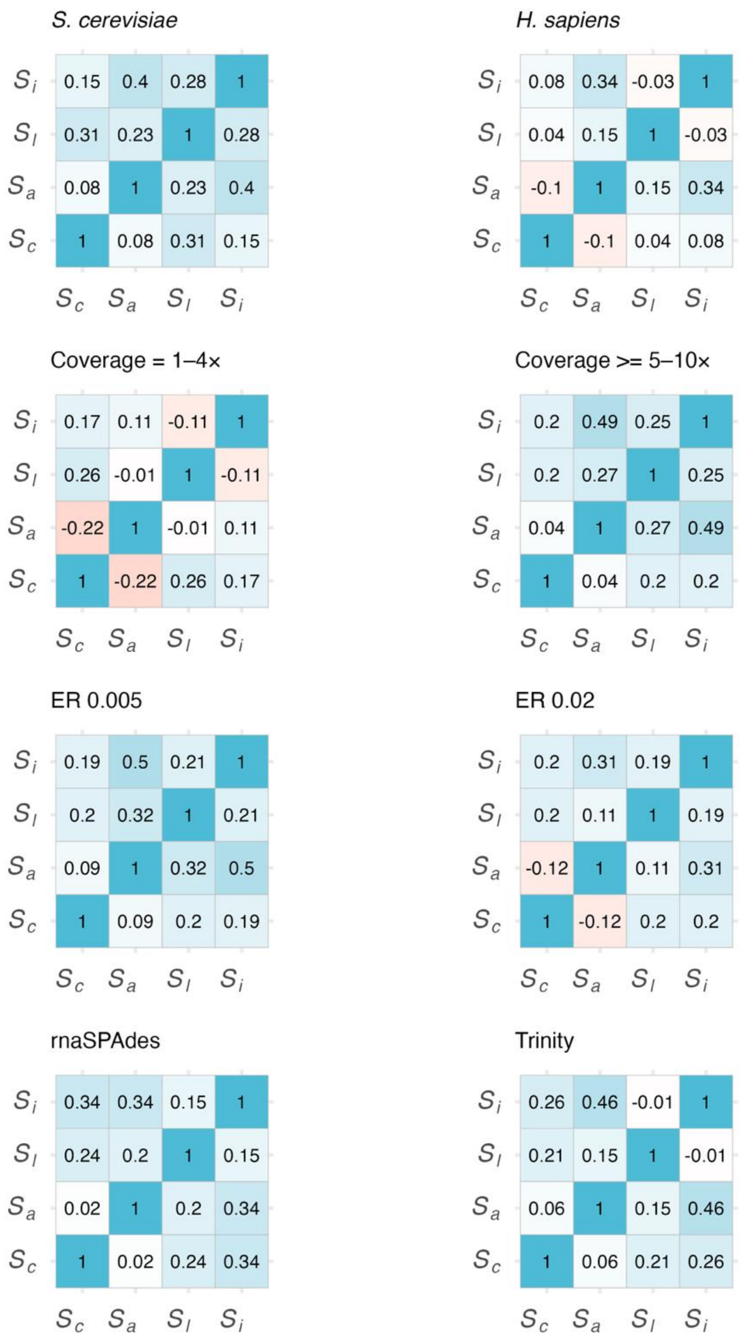

Supplementary Figure 1. Median pairwise correlation coefficients between CATS-rf score components across assemblies stratified by selected species, coverage levels, sequencing error rates, and assemblers. Group sizes were as follows: *Saccharomyces cerevisiae*, n = 84; *Homo sapiens*, n = 84; coverage 1–4×, n = 72; coverage ≥5–10×, n = 432; ER = 0.005, n = 168; ER = 0.02, n = 168; rnaSPAdes, n = 126; Trinity, n = 126. S_c = coverage component, S_a = accuracy component, S_l = local fidelity component, S_i = integrity component, ER = error rate.

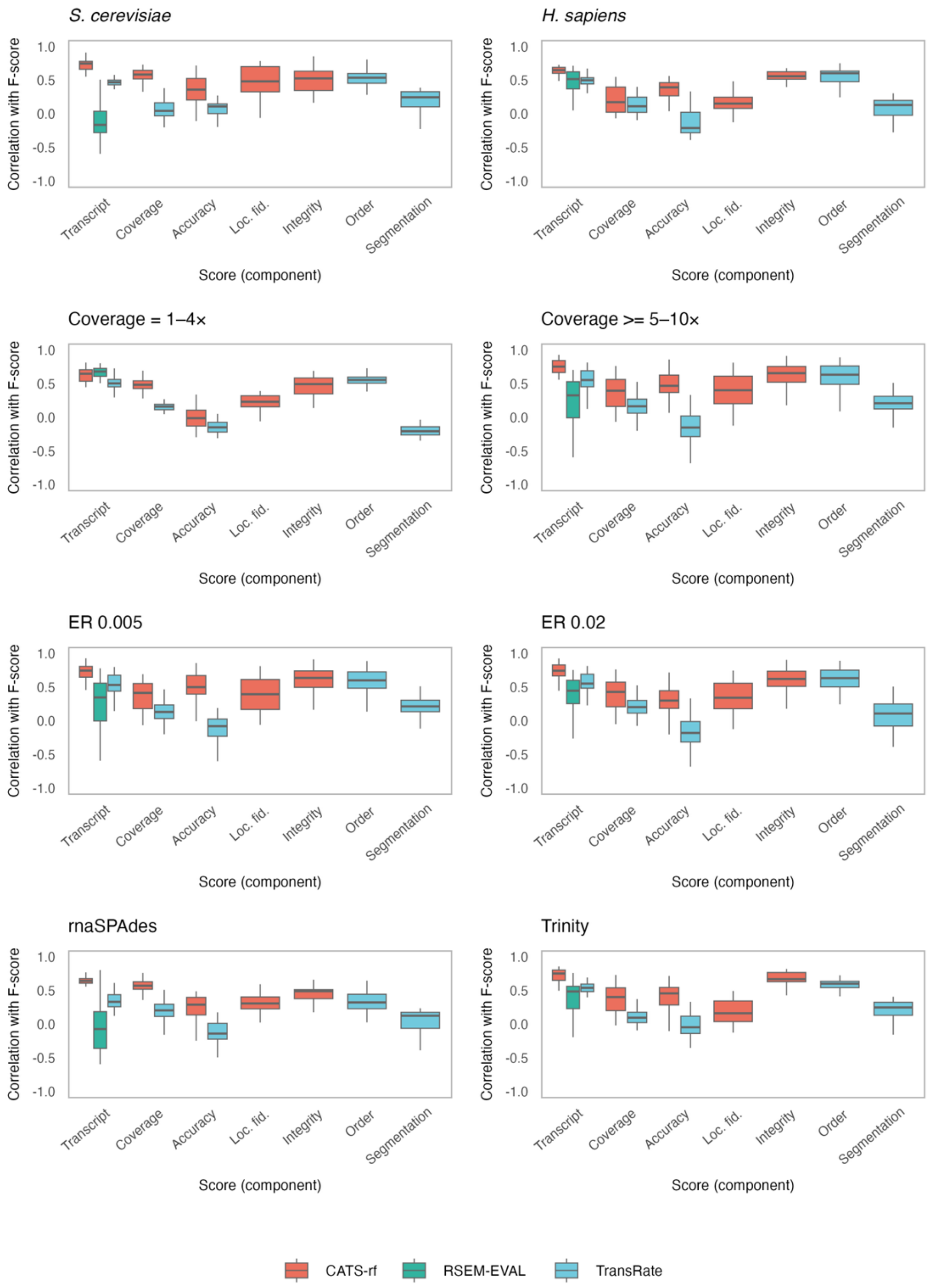

Supplementary Figure 2. Distribution of Spearman correlation coefficients between transcript F-scores and transcript scores from CATS-rf, RSEM-EVAL, and TransRate across assemblies stratified by selected species, coverage levels, sequencing error rates, and assemblers. Group sizes were as follows: *Saccharomyces cerevisiae*, n = 84; *Homo sapiens*, n = 84; coverage 1–4×, n = 72; coverage ≥5–10×, n = 432; ER = 0.005, n = 168; ER = 0.02, n = 168; rnaSPAdes, n = 126; Trinity, n = 126. Boxplots represent the median and IQR, with whiskers extending to ±1.5 × IQR. colors indicate transcriptome quality assessment tools: red = CATS-rf, green = RSEM-EVAL, light blue = TransRate. ER = error rate.

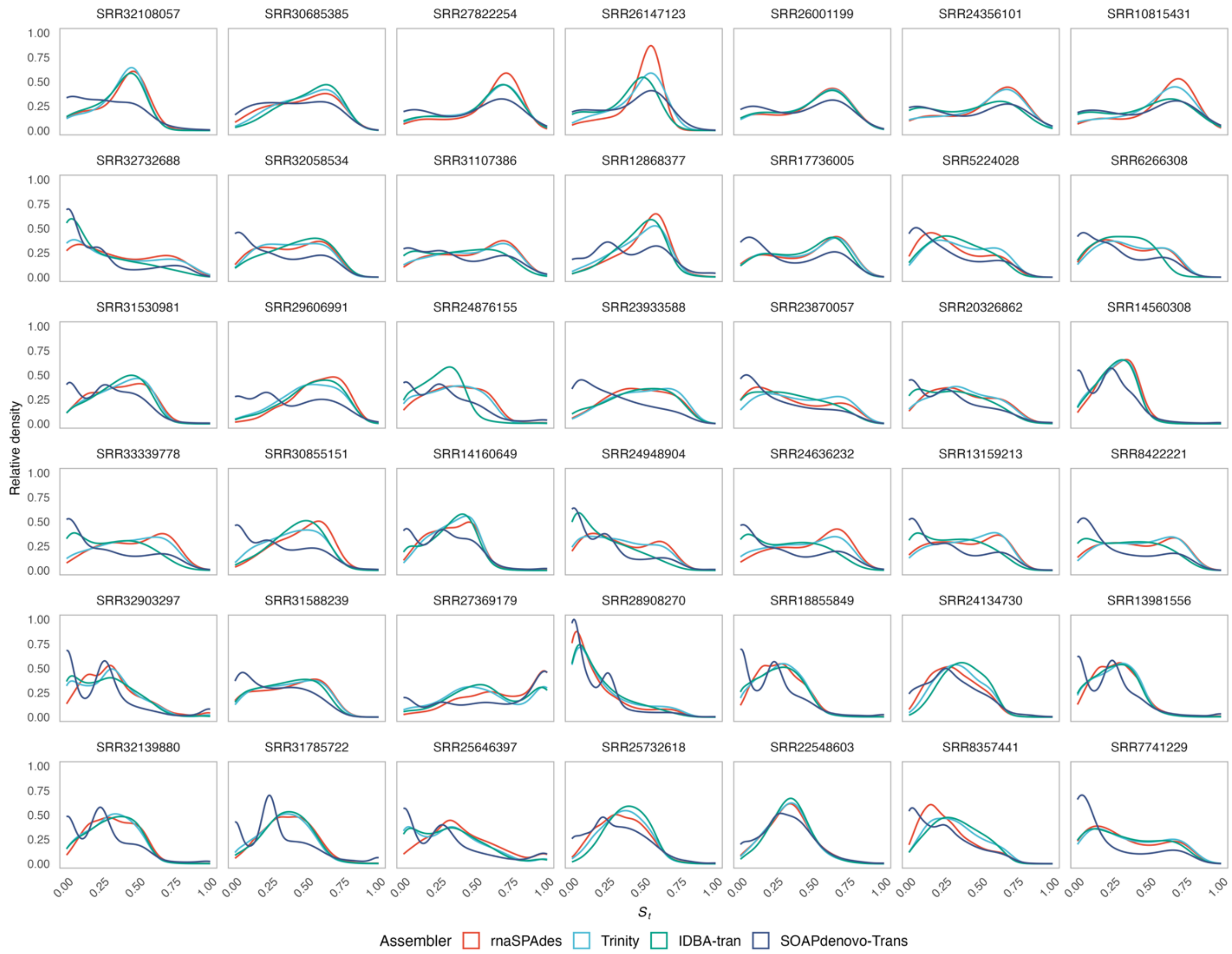

Supplementary Figure 3. Distribution of CATS-rf transcript scores in assemblies generated from public RNA-seq libraries, labeled with corresponding Sequence Read Archive accession numbers. Colors indicate assemblers: red = rnaSPAdes, light blue = Trinity, green = IDBA-tran, dark blue = SOAPdenovo-Trans S_t = CATS-rf transcript score.

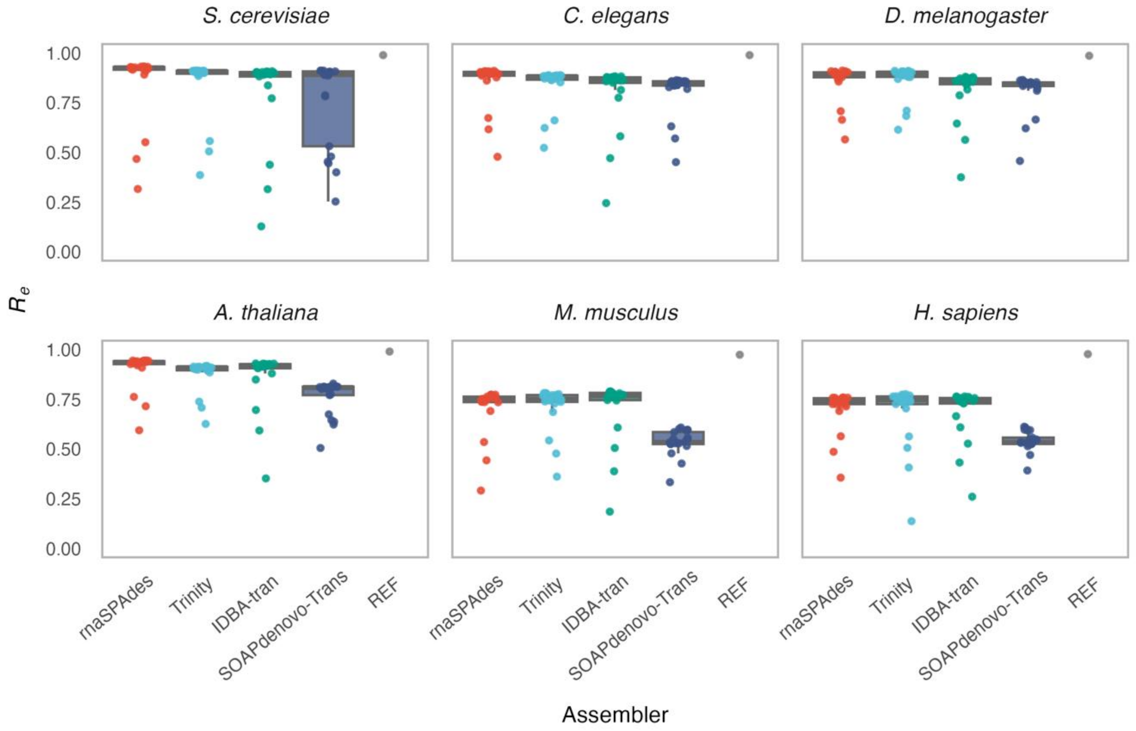

Supplementary Figure 4. Distribution of relative CATS-rb exon scores across species and assemblers in controlled simulations (*n* = 21 assemblies per group). Colors indicate assemblers: red = rnaSPAdes, light blue = Trinity, green = IDBA-tran, dark blue = SOAPdenovo-Trans; grey indicates the reference transcriptome. R_e = relative exon score.

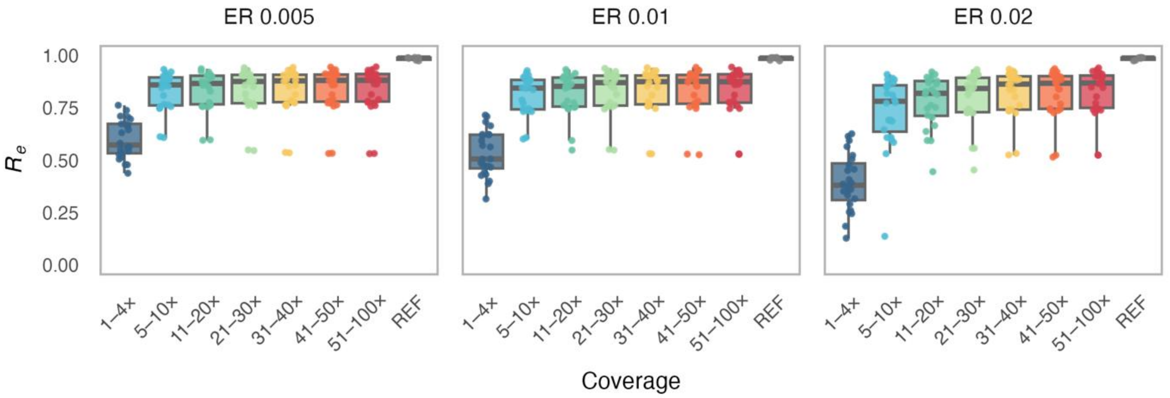

Supplementary Figure 5. Distribution of relative CATS-rb exon scores by coverage and error rate in controlled simulations (*n* = 24 assemblies per group, *n* = 6 reference transcriptomes). Colors denote coverage: dark blue = 1–4×, light blue = 5–10×, green = 11–20×, brown = 21–30×, yellow = 31–40×, orange = 41–50×, red = 51–100×; grey indicates the reference transcriptome. R_e = relative exon score.

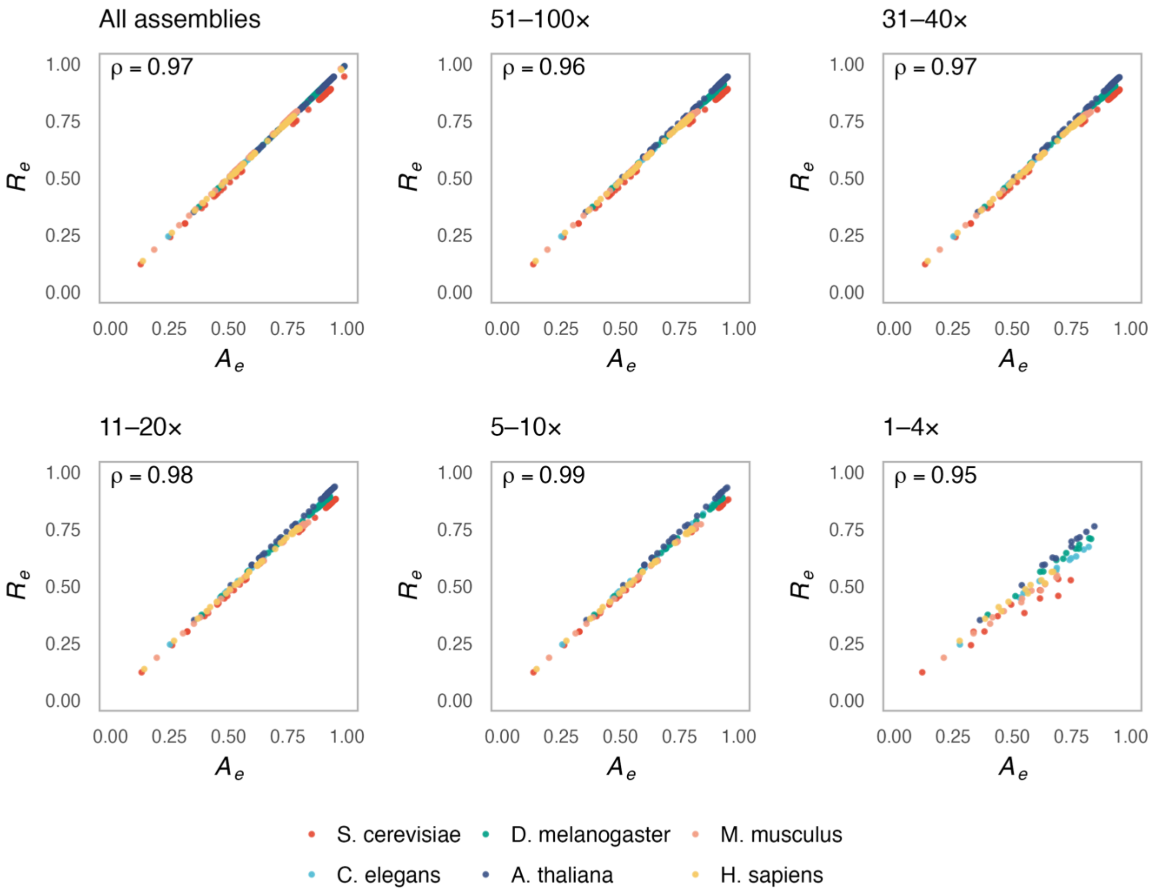

Supplementary Figure 6. Correlation between CATS-rb relative and annotation-based exon scores in assemblies with decreasing coverage (indicated in subplot titles). Colors represent species: red = *Saccharomyces cerevisiae*, light blue = *Caenorhabditis elegans*, green = *Drosophila melanogaster*, dark blue = *Arabidopsis thaliana*, salmon = *Mus musculus*, yellow = *Homo sapiens*. R_e = relative exon score, A_e = annotation-based exon score, ρ = Spearman correlation coefficient.

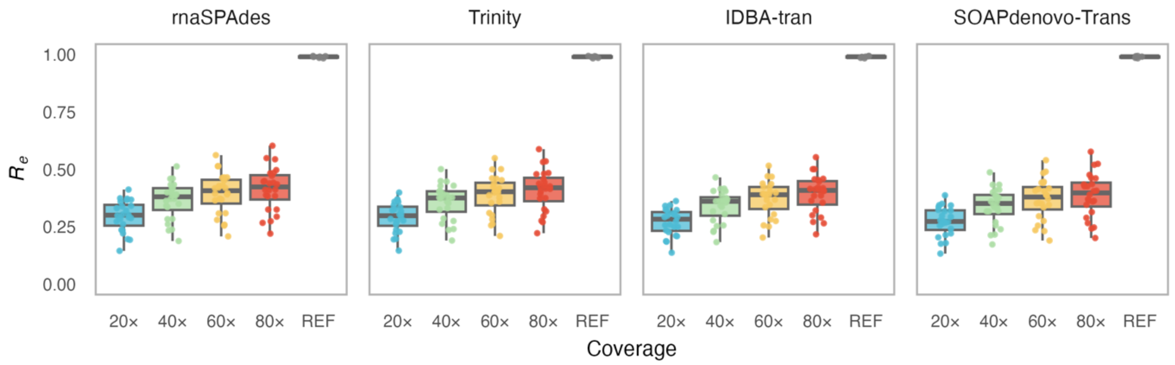

Supplementary Figure 7. Distribution of relative CATS-rb exon scores across assemblers and coverage in realistically simulated libraries (n = 24 assemblies per group, n = 6 reference transcriptomes). Colors represent coverage: light blue = 20×, green = 40×, yellow = 60×, red = 80×; grey indicates the reference transcriptome. R_e = relative exon score.

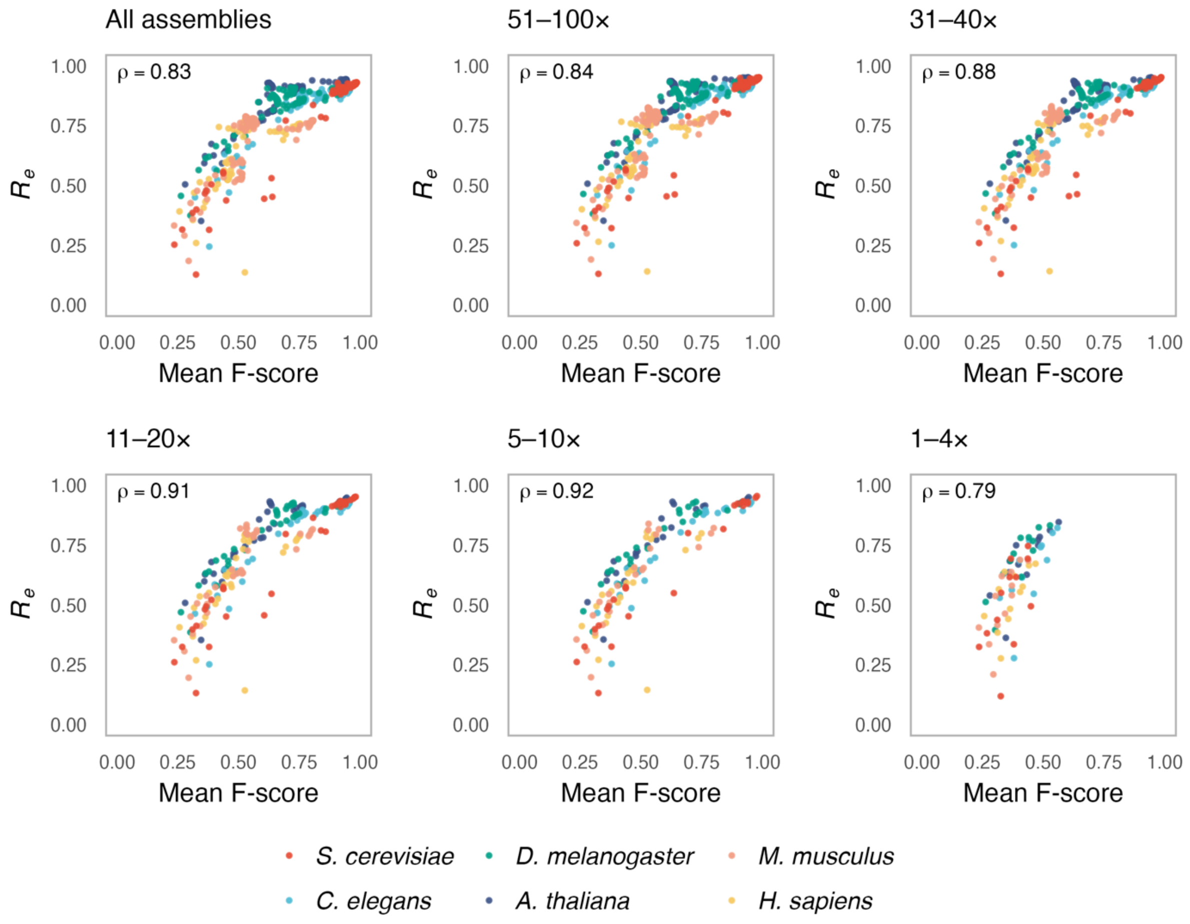

Supplementary Figure 8. Correlation between CATS-rb relative exon scores and mean transcript F-scores across assemblies with decreasing coverage (indicated in subplot titles). Colors represent species: red = *Saccharomyces cerevisiae*, light blue = *Caenorhabditis elegans*, green = *Drosophila melanogaster*, dark blue = *Arabidopsis thaliana*, salmon = *Mus musculus*, yellow = *Homo sapiens*. R_e = relative exon score, ρ = Spearman correlation coefficient.

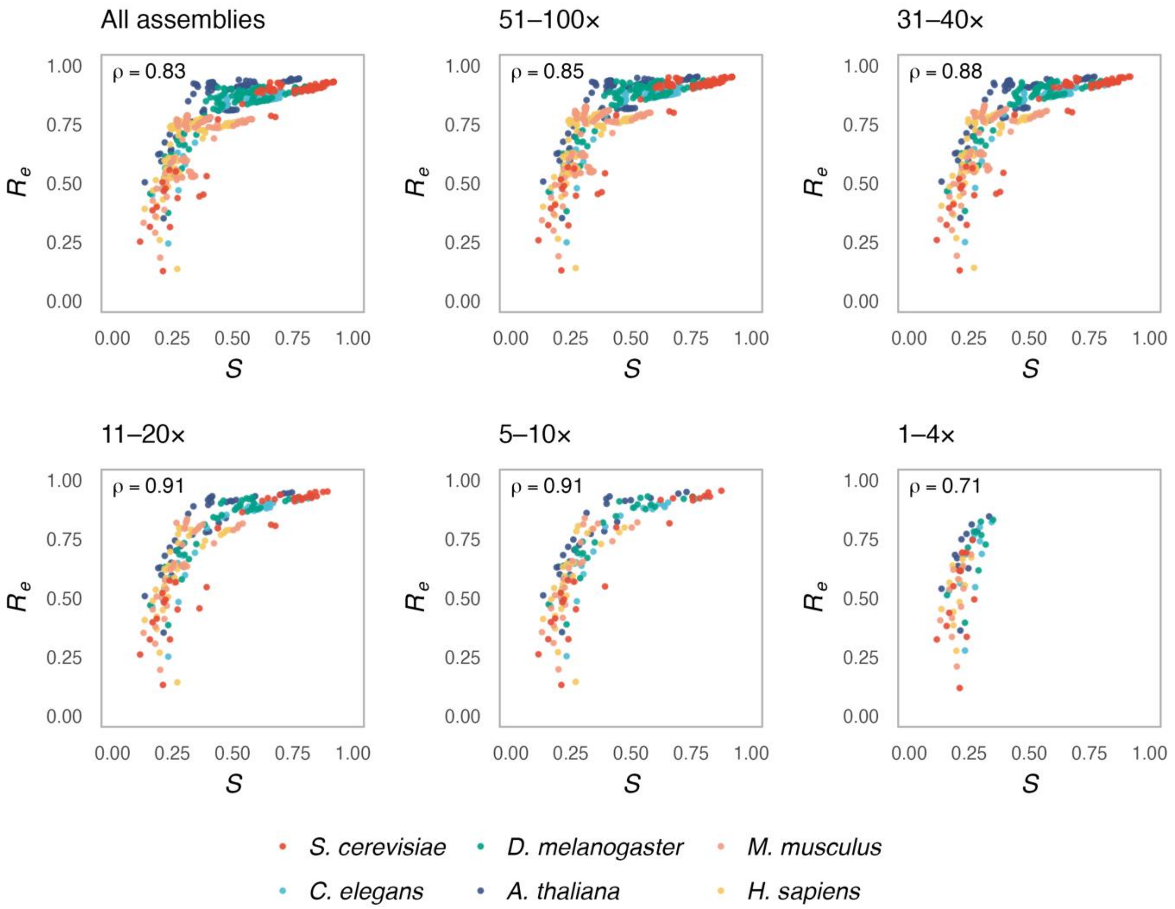

Supplementary Figure 9. Correlation between CATS-rb relative exon scores and CATS-rf assembly scores across assemblies with decreasing coverage (indicated in subplot titles). Colors represent species: red = *Saccharomyces cerevisiae*, light blue = *Caenorhabditis elegans*, green = *Drosophila melanogaster*, dark blue = *Arabidopsis thaliana*, salmon = *Mus musculus*, yellow = *Homo sapiens*. R_e = relative exon score, S = CATS-rf assembly score, ρ = Spearman correlation coefficient.

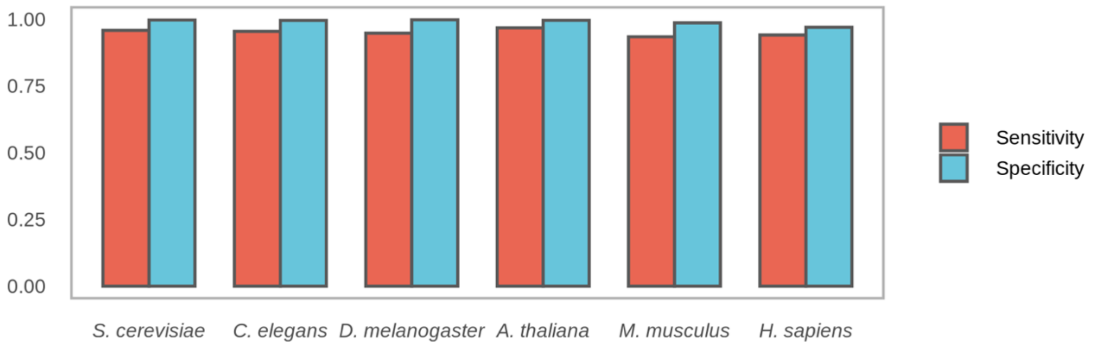

Supplementary Figure 10. Performance of CATS-rb in classifying chimeric transcripts as structurally inconsistent. Colors denote performance metrics: red = sensitivity, light blue = specificity.

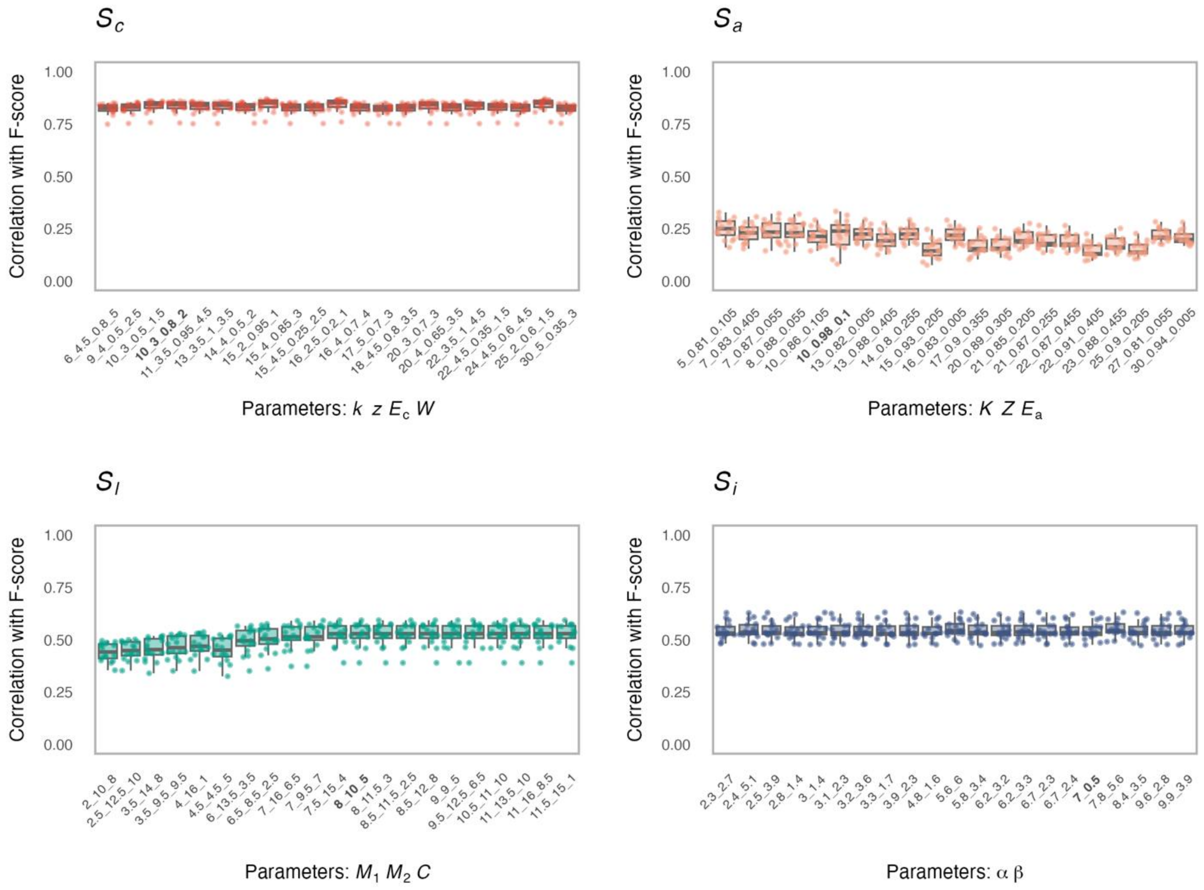

Supplementary Figure 11. Distribution of correlation coefficients between CATS-rf score components and transcript F-scores across CATS-rf runs on n = 16 assemblies with parameter sets sampled via random grid-search (x-axis). Default parameter values are shown in bold. Colors denote score components associated with each parameter set: red = coverage component, salmon = accuracy component, green = local fidelity component, dark blue = integrity component. S_c = coverage component, S_a = accuracy component, S_l = local fidelity component, S_i = integrity component, k = rolling window length for LCR detection, z = mean rolling window coverage threshold for LCR definition, E_c = LCR extension penalty, W = base coverage weight, K = rolling window length for LAR detection, Z = mean rolling window accuracy threshold, E_a = LAR extension penalty, M_1 = lower multiplicative factor, M_2 = higher multiplicative factor, C = correction factor, α = compression factor 1, β = compression factor 2.

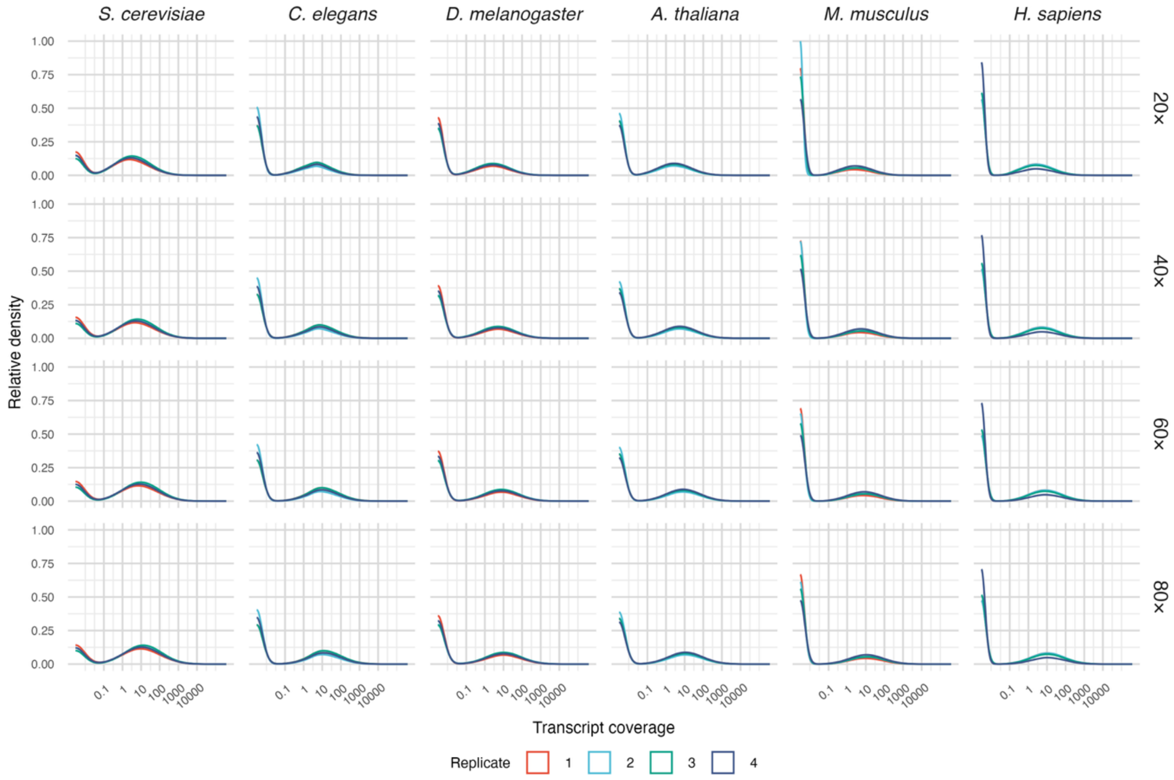

Supplementary Figure 12. Reference transcript coverage distribution in realistically simulated datasets. Colors indicate library replicates: red = Replicate 1, light blue = Replicate 2, green = Replicate 3, dark blue = Replicate 4.

Supplementary Table 1. In-depth comparison between CATS-rf and TransRate algorithms (1).

Category	CATS-rf	TransRate (read-metric mode)
Read mapping	Bowtie2 (global mode, sensitive preset)<br>N alignments per read: 10 (adjustable)<br>Mismatch penalty: 2<br>ED threshold: /<br>ED threshold for secondary mappings:<br>ED (primary) + 10% read length (adjustable)	SNAP<br>N alignments per read: 10<br>Mismatch penalty: /<br>ED threshold: 30<br>ED threshold for secondary mappings:<br>ED (primary) + 5
Multimapped read assignment	Each read is assigned to a single transcript<br>Assignment probability is proportional to transformed TPM<br>TPM is calculated by Kallisto<br>Enforces co-assignment of paired reads	Each read is assigned to a single transcript<br>Assignment probability is proportional to TPM<br>TPM is calculated by Salmon
Coverage metrics	N, % of covered bases per transcript and assembly<br>N, % of completely covered and uncovered transcripts<br>Mean per-base coverage per transcript<br>N, % of bases with coverage higher than adjustable thresholds<br>Maximum uncovered region length per transcript<br>Mean coverage of transcript ends<br>N, % of bases in LCRs per transcript and assembly<br>LCR length per transcript<br>Coverage metric plots	N, % of uncovered bases per transcript<br>N, % of transcripts with at least one uncovered base<br>N, % of transcripts with mean coverage < 1<br>N, % of transcripts with mean coverage < 10
Coverage score component	Coverage penalty is assigned to each LCR proportionally to coverage reduction and LCR length<br>Coverage component is defined as the complement of coverage penalty sum normalized by transcript length	Proportion of uncovered bases per transcript
Accuracy metrics	% of accurate bases per transcript<br>N, % of bases with accuracy higher than adjustable thresholds<br>N, % of bases in LARs per transcript and assembly<br>LAR length per transcript<br>Accuracy metric plots	/
Accuracy score component	Accuracy penalty is assigned to each LAR proportionally to accuracy reduction and LAR length<br>Accuracy component is defined as the complement of accuracy penalty sum normalized by transcript length	Mean ED of read mappings per transcript normalized by the maximum allowed ED
Paired-end read metrics	N, % of reads with pair not mapped to the assembly<br>N, % of reads with pair mapped in an unexpected orientation<br>N, % of reads with pair mapped too far<br>N, % of improperly paired reads within a transcript per transcript and assembly<br>N, % of reads with pair mapped to another transcript<br>N, % of fragmented transcripts<br>N, % of reads representing bridging events on transcript ends per transcript and assembly<br>Paired-end read metric plots	N, % of reads with pair mapped to another transcript<br>N, % of fragmented transcripts
Local fidelity score component	Incorporates the number of reads whose pairs fail to map to the assembly, the number of reads mapping in unexpected orientations, and a pair distance penalty scaled according to inter-pair distance and the fragment length distribution<br>The local fidelity component normalizes the combined contribution of these metrics by the total number of mapped reads	/
Integrity score component	Read pairs mapping to different transcripts incur a bridge penalty, scaled by their distance to transcript ends<br>The bridge index further adjusts this penalty based on the total number of mapped reads<br>Integrity component applies a sigmoid transformation to the bridge penalty, enhancing sensitivity to fragmentation signals	/
Order score	/	Proportion of improperly paired reads per transcript<br>Incorporates reads with unmapped pairs, reads with pairs mapped to other transcripts, improperly oriented reads, and read pairs mapping too far
Segmentation score	/	Identifies chimeric transcripts using coverage profiles
Transcript score	Product of score components	Product of score components
Assembly score	Mean of transcript scores, evaluates quality of assembled transcripts. Proportion of mapped reads is reported as a separate metric	Product of the geometric mean of transcript scores and the proportion of mapped reads, evaluates quality and completeness relative to the input reads

SNAP = scalable nucleotide alignment program, ED = edit distance, TPM = transcripts per million, LCR = low-coverage region, LAR = low-accuracy region

Supplementary Table 2. Default values of tunable parameters for CATS-rf score components.

Score component	Feature	Parameter	Default value	Rationale
Coverage component (S_c)	Low-coverage region (LCR)	Rolling window length for LCR detection (bp)	10	LCRs are identified by calculating the mean per-base coverage within rolling windows of a fixed length and merging all contiguous bases whose corresponding window-mean coverage falls below a specified threshold. The default length and threshold values were selected to distinguish regions with consistently weak read support from isolated single-base coverage drops indicative of read indels or transcript variation
Coverage component (S_c)	Low-coverage region (LCR)	Mean rolling window coverage threshold for LCR definition	3	
Coverage component (S_c)	Coverage penalty (P_c)	LCR Extension penalty (E_c)	0.5	E_c controls the effect of LCR length on the coverage penalty. The default value was chosen to apply a moderate penalty for increased LCR length relative to per-base coverage. Specifically, extending the LCR by one base increases the total coverage penalty equivalently to adding two bases with zero coverage
Coverage component (S_c)	Per-base coverage function ($f(C_n)$)	Base coverage weight (W)	1.5	W controls the effect of per-base coverage values on coverage penalty by scaling $f(C_n)$. The default value moderately reduces the penalty assigned to non-zero coverage values
Accuracy component (S_a)	Low-accuracy region (LAR)	Rolling window length for LAR detection (bp)	10	LARs are defined by computing the mean per-base accuracy within rolling windows of a fixed length and merging all contiguous bases with the corresponding window-mean accuracy below a specified threshold. The default length and threshold values were chosen to detect extended regions of reduced accuracy indicative of misassembly, while avoiding the penalization of sporadic sequencing errors that occur at standard short-read error rates and low-to-medium transcript coverage
Accuracy component (S_a)	Low-accuracy region (LAR)	Mean rolling window accuracy threshold for LAR definition	0.98	
Accuracy component (S_a)	Accuracy penalty (P_a)	LAR Extension penalty (E_a)	0.1	E_a controls the effect of LAR length on the accuracy penalty. The default value was selected to incur a moderate penalty for increased LAR length relative to per-base accuracy. Specifically, extending the LAR by one base increases the total accuracy penalty equivalently to adding five bases with 0.5 accuracy
Local fidelity component (S_l)	Lower distance threshold (D_1)	Lower multiplicative factor (M_1)	8	M_1 is a scaling factor applied to D_1, which defines the threshold for inter-pair read distance used to determine whether a read pair should be penalized. D_1 is calculated using a standard IQR-based outlier criterion, where M_1 controls how many IQRs beyond Q_3 a read-pair distance must fall to be considered an outlier. The default value of M_1 was selected to minimize false-positive penalties resulting from variation in fragment length distributions across libraries
Local fidelity component (S_l)	Higher distance threshold (D_2)	Higher multiplicative factor (M_2)	10	M_2 scales D_2, which maps the distance penalty for read-pair outliers to the range [0, 1]. Its default value corresponds to a 2-IQR extension relative to D_1, such that outliers between 8 and 10 IQRs beyond Q_3 are scaled from 0 to 1, while stronger outliers receive the maximum penalty
Local fidelity component (S_l)	Lower and higher distance thresholds (D_1 and D_2)	Correction factor (C)	5	C is an additive constant applied to D_1 and D_2 to ensure robust thresholding in libraries containing a substantial proportion of overlapping read pairs. The default value of 5 was selected to provide minimal inflation of D_1 and D_2 while maintaining appropriate distance penalization in datasets with high levels of read-pair overlap
Integrity component (S_i)	Integrity component (S_i)	Compression factor 1 (α)	7	α and β represent compression factors of the sigmoid transformation applied to the bridge index. The transformation is applied to compensate for consistent mapping of paired reads to interior transcript regions, which weaken the fragmentation signal. The default settings were chosen to provide a balanced sigmoid shape which is sufficiently steep to penalize fragmentation, yet stable across unfragmented transcripts
Integrity component (S_i)	Integrity component (S_i)	Compression factor 2 (β)	0.5	

Supplementary Table 3. Reference transcriptome assemblies used in simulating RNA-seq reads for CATS benchmarking. The final transcriptome was generated by merging the coding and non-coding reference transcriptomes.

**Species**	**Build**	**Ensembl release**	**N transcripts**	**Total length (Mb)**
S. cerevisiae	R64-1-1	111	7036	8.9
C. elegans	WBcel235	111	60000	58.2
D. melanogaster	BDGP6.46	111	35722	92.8
A. thaliana	TAIR10	56	54013	89.2
M. musculus	GRCm39	111	145855	243.9
H. sapiens	GRCh38	111	275921	471.4

Supplementary Table 4. Number of expressed transcripts in realistically simulated RNA-seq libraries used for CATS benchmarking.

Species	Replicate	N (%) expressed transcripts
S. cerevisiae	1	5136 (73%)
	2	5558 (79%)
	3	5981 (85%)
	4	5558 (79%)
C. elegans	1	30000 (50%)
	2	21000 (35%)
	3	30000 (50%)
	4	25200 (42%)
D. melanogaster	1	12146 (34%)
	2	15361 (43%)
	3	15718 (44%)
	4	13932 (39%)
A. thaliana	1	21605 (40%)
	2	18364 (34%)
	3	21605 (40%)
	4	23766 (44%)
M. musculus	1	30630 (21%)
	2	37922 (26%)
	3	39381 (27%)
	4	49591 (34%)
H. sapiens	1	110368 (40%)
	2	110368 (40%)
	3	99312 (36%)
	4	63462 (23%)

Supplementary Table 5. Number of reads in realistically simulated RNA-seq libraries used for CATS benchmarking.

		**N reads (million)**			
**Species**	**Coverage**	**Replicate 1**	**Replicate 2**	**Replicate 3**	**Replicate 4**
S. cerevisiae	20×	0.6	0.7	0.7	0.7
	40×	1.3	1.4	1.5	1.4
	60×	1.9	2.1	2.2	2.1
	80×	2.6	2.8	3.0	2.8
C. elegans	20×	2.9	2.0	3.0	2.5
	40×	5.8	4.0	5.9	4.9
	60×	8.7	6.0	8.8	7.4
	80×	11.6	8.0	11.8	9.8
D. melanogaster	20×	3.2	4.0	4.1	3.6
	40×	6.4	8.0	8.2	7.2
	60×	9.6	11.9	12.3	10.8
	80×	12.8	15.9	16.5	14.4
A. thaliana	20×	3.6	3.0	3.6	4.0
	40×	7.1	6.1	7.1	7.9
	60×	10.7	9.1	10.7	11.8
	80×	14.3	12.2	14.3	15.8
M. musculus	20×	5.1	6.3	6.6	8.3
	40×	10.2	12.6	13.2	16.5
	60×	15.3	18.9	19.8	24.8
	80×	20.5	25.2	26.4	33.0
H. sapiens	20×	18.7	18.9	17.0	10.8
	40×	37.4	37.8	34.0	21.7
	60×	56.1	56.6	50.9	32.5
	80×	74.9	75.4	68.0	43.3

Supplementary Table 6. Public RNA-seq libraries used in CATS benchmarking.

SRA accession	Species	Publication date (DD.MM.YYYY)	Mean read length (bp)	N read pairs (million)	Total size (Gb)
SRR32108057	*S. cerevisiae*	24.01.2025	150	27.2	8.2
SRR30685385	*S. cerevisiae*	17.09.2024	72	2.2	0.3
SRR27822254	*S. cerevisiae*	26.03.2024	51	17.9	1.8
SRR26147123	*S. cerevisiae*	31.12.2023	146	7.0	2.0
SRR26001199	*S. cerevisiae*	11.11.2023	74	5.5	0.8
SRR24356101	*S. cerevisiae*	29.04.2023	150	19.8	1.8
SRR10815431	*S. cerevisiae*	21.02.2020	150	3.7	0.4
SRR32732688	*C. elegans*	17.03.2025	151	31.1	9.4
SRR32058534	*C. elegans*	21.01.2025	150	27.6	8.3
SRR31107386	*C. elegans*	20.01.2025	150	26.4	7.9
SRR12868377	*C. elegans*	10.02.2022	150	60.7	18.2
SRR17736005	*C. elegans*	25.01.2022	50	35.1	3.6
SRR5224028	*C. elegans*	01.03.2018	100	36.2	7.2
SRR6266308	*C. elegans*	25.02.2018	125	27.1	6.8
SRR31530981	*D. melanogaster*	28.11.2024	101	28.6	5.8
SRR29606991	*D. melanogaster*	28.06.2024	151	31.9	9.6
SRR24876155	*D. melanogaster*	17.11.2023	151	30.4	10.4
SRR23933588	*D. melanogaster*	27.06.2023	51	87.8	9.0
SRR23870057	*D. melanogaster*	17.03.2023	80	45.3	7.2
SRR20326862	*D. melanogaster*	25.07.2022	150	32.9	9.9
SRR14560308	*D. melanogaster*	01.01.2022	150	19.7	5.8
SRR33339778	*A. thaliana*	30.04.2025	150	37.6	11.3
SRR30855151	*A. thaliana*	11.10.2024	147	27.9	8.2
SRR14160649	*A. thaliana*	12.11.2023	101	37.5	7.6
SRR24948904	*A. thaliana*	20.10.2023	150	44.0	13.2
SRR24636232	*A. thaliana*	08.10.2023	150	31.0	9.3
SRR13159213	*A. thaliana*	31.12.2021	101	43.7	8.8
SRR8422221	*A. thaliana*	23.01.2019	74	33.0	4.9
SRR32903297	*M. musculus*	28.03.2025	150	28.3	8.5
SRR31588239	*M. musculus*	03.12.2024	50	41.0	4.1
SRR27369179	*M. musculus*	11.07.2024	151	66.2	20.0
SRR28908270	*M. musculus*	07.06.2024	101	62.9	12.7
SRR18855849	*M. musculus*	01.10.2023	151	49.9	15.1
SRR24134730	*M. musculus*	15.08.2023	100	7.4	3.5
SRR13981556	*M. musculus*	15.10.2021	151	28.3	8.5
SRR32139880	*H. sapiens*	27.01.2025	150	25.9	7.8
SRR31785722	*H. sapiens*	30.12.2024	150	71.8	21.5
SRR25646397	*H. sapiens*	13.06.2024	150	45.7	13.7
SRR25732618	*H. sapiens*	13.12.2023	101	50.5	10.2
SRR22548603	*H. sapiens*	21.12.2022	101	53.8	10.9
SRR8357441	*H. sapiens*	02.06.2019	100	51.7	10.3
SRR7741229	*H. sapiens*	26.03.2019	51	40.5	4.1

Supplementary Table 7. Reference genomes and the corresponding genomic annotation used in CATS-rb benchmarking. The genomic annotations included both coding and non-coding genes.

**Species**	**Build**	**Ensembl release**	**N scaffolds**	**Total length (Mb)**
S. cerevisiae	R64-1-1	111	17	12.2
C. elegans	WBcel235	111	7	100.3
D. melanogaster	BDGP6.46	111	1870	143.7
A. thaliana	TAIR10	56	7	119.7
M. musculus	GRCm39	111	61	2728.2
H. sapiens	GRCh38	111	194	3099.8

Supplementary Table 8. Species-specific CATS-rb parameters used in benchmarking.

Species	Max gene size (bp)	Spaln species preset	Min exon identity proportion	Min exon length (bp)	Max intron length (bp)	Min exon set length (bp)	Min transcript set length (bp)	Max transcript set length (bp)
S. cerevisiae	25000	sacccere	0.98	30	1500	0	100	25000
C. elegans	100000	caeneleg	0.98	30	20000	0	150	100000
D. melanogaster	500000	drosmela	0.98	30	90000	0	200	500000
A. thaliana	50000	arabthal	0.98	30	5000	0	100	50000
M. musculus	3000000	mus_musc	0.998	30	600000	125	450	3000000
H. sapiens	3000000	homosapi	0.998	30	400000	100	300	3000000

Supplementary Methods

Random Grid-Search Analysis of CATS-rf Parameters

To evaluate the impact of parameter variation on CATS-rf score components, we performed a random grid-search analysis using 16 assemblies derived from the first replicate of realistic simulations. The dataset comprised rnaSPAdes assemblies from four species (*S. cerevisiae*, *C. elegans*, *D. melanogaster,* and *A. thaliana*), each generated at four mean coverage levels (20×, 40×, 60×, and 80×). Each assembly was evaluated using CATS-rf with defult and 20 randomly sampled parameter combinations defined by the grid search. Correlation between individual CATS-rf score components and transcript F-scores were computed within each assembly. Parameters were varied using the following ranges and step sizes: rolling window length for LCR detection (5–30, step 1), mean rolling window coverage threshold for LCR definition (1.5–5, step 0.5), LCR extension penalty (0.1–1, step 0.05), base coverage weight (1–5, step 0.5), rolling window length for LAR detection (5–30, step 1), mean rolling window accuracy threshold (0.8–1, step 0.01), LAR extension penalty (0.005–0.5, step 0.005), lower multiplicative factor (2–12, step 0.5), higher multiplicative factor (4–16, step 0.5), correction factor (1–10, step 1), compression factor 1 (2–10, step 0.5), and compression factor 2 (0.5–7, step 0.5). Results were analysed using a custom R script (R version 4.4.2) (2), with the following packages used for data manipulation and visualization: data.table (version 1.16.4) (3), ggplot2 (version 3.5.1) (4).

Supplementary References

1. Smith-Unna, R., Boursnell, C., Patro, R., Hibberd, J. M. & Kelly, S. TransRate: reference-free quality assessment of de novo transcriptome assemblies. *Genome Res.* **26** (8), 1134-1144 (2016).
2. R Core Team. R: A language and environment for statistical computing. R Foundation for Statistical Computing, Vienna, Austria. Available from https://www.R-project.org/ (Accessed March 17, 2026).
3. Barrett, T. et al. data.table: Extension of `data.frame`. Available from https://CRAN.R-project.org/package=data.table (Accessed March 17, 2026).
4. Wickham, H. ggplot2: Elegant Graphics for Data Analysis. Springer-Verlag New York, 2016.
