# Supplementary material for: Comprehensive assessment of transcriptome assembly quality using CATS
Source: Nat Commun. 2026 Apr 20;17:5419. doi: 10.1038/s41467-026-72171-8 (PMC13280353; doi:10.1038/s41467-026-72171-8)
Supplement: Supplementary file 2 — Reporting Summary [file 41467_2026_72171_MOESM2_ESM.pdf]

nature portfolio

Corresponding author(s): Kristian Vlahoviček

Last updated by author(s): Mar 25, 2026

Reporting Summary

Nature Portfolio wishes to improve the reproducibility of the work that we publish. This form provides structure for consistency and transparency in reporting. For further information on Nature Portfolio policies, see our Editorial Policies and the Editorial Policy Checklist.

Statistics

For all statistical analyses, confirm that the following items are present in the figure legend, table legend, main text, or Methods section.

n/a	Confirmed	
☐	☒	The exact sample size (*n*) for each experimental group/condition, given as a discrete number and unit of measurement
☐	☒	A statement on whether measurements were taken from distinct samples or whether the same sample was measured repeatedly
☐	☒	The statistical test(s) used AND whether they are one- or two-sided<br>*Only common tests should be described solely by name; describe more complex techniques in the Methods section.*
☐	☒	A description of all covariates tested
☒	☐	A description of any assumptions or corrections, such as tests of normality and adjustment for multiple comparisons
☐	☒	A full description of the statistical parameters including central tendency (e.g. means) or other basic estimates (e.g. regression coefficient) AND variation (e.g. standard deviation) or associated estimates of uncertainty (e.g. confidence intervals)
☐	☒	For null hypothesis testing, the test statistic (e.g. *F*, *t*, *r*) with confidence intervals, effect sizes, degrees of freedom and *P* value noted<br>*Give P values as exact values whenever suitable.*
☒	☐	For Bayesian analysis, information on the choice of priors and Markov chain Monte Carlo settings
☒	☐	For hierarchical and complex designs, identification of the appropriate level for tests and full reporting of outcomes
☐	☒	Estimates of effect sizes (e.g. Cohen's *d*, Pearson's *r*), indicating how they were calculated

Our web collection on statistics for biologists contains articles on many of the points above.

Software and code

Policy information about availability of computer code

Data collection	RNA-seq datasets were simulated with Polyester v1.32.0 (R v4.4.2) or obtained from the Sequence Read Archive (SRA) via SRA Toolkit (v3.2.0).
Data analysis	Analyses were performed with: Trim Galore v0.6.1, cutadapt v4.6, rnaSPAdes v3.15.4, Trinity v2.15.1, IDBA-tran v1.1.1, SOAPdenovo-Trans v1.0.4, CATS-rf v1.0.0, RSEM-EVAL v1.11, TransRate v1.0.4, kallisto v0.50.1, CRB-BLAST v1.0.0, BLAT v2.17.0, CATS-rb v1.0.0, and R v4.4.2 with the following packages: Biostrings v2.27.1, cocor v1.1.4, DescTools v0.99.60, data.table v1.16.4, ggplot2 v3.5.1, ggcorrplot v0.1.4.1, cowplot v1.1.3, and ggbiplot v0.6.2.<br>CATS-rf source code is available at https://github.com/bodulic/CATS-rf<br>CATS-rb source code is available at https://github.com/bodulic/CATS-rb<br>Custom benchmarking scripts are available at https://github.com/bodulic/CATS_benchmark

For manuscripts utilizing custom algorithms or software that are central to the research but not yet described in published literature, software must be made available to editors and reviewers. We strongly encourage code deposition in a community repository (e.g. GitHub). See the Nature Portfolio guidelines for submitting code & software for further information.

Data

Policy information about availability of data

All manuscripts must include a data availability statement. This statement should provide the following information, where applicable:

- Accession codes, unique identifiers, or web links for publicly available datasets
- A description of any restrictions on data availability
- For clinical datasets or third party data, please ensure that the statement adheres to our policy

All datasets analyzed in this study are publicly available. Reference transcriptome assemblies, genome sequences, and gene annotations used in RNA-seq library simulation and CATS benchmarking were obtained from Ensembl. Reference data for S. cerevisiae (R64-1-1), C. elegans (WBcel235), D. melanogaster (BDGP6.46), M. musculus (GRCm39), and H. sapiens (GRCh38) were downloaded from Ensembl release 111. Reference data for A. thaliana (TAIR10) were obtained from Ensembl Plants release 56.

Public RNA-seq libraries used in CATS benchmarking are available via SRA under the following accession numbers: SRR32108057, SRR30685385, SRR27822254, SRR26147123, SRR26001199, SRR24356101, SRR10815431 (S. cerevisiae); SRR32732688, SRR32058534, SRR31107386, SRR12868377, SRR17736005, SRR5224028, SRR6266308 (C. elegans); SRR31530981, SRR29606991, SRR24876155, SRR23933588, SRR23870057, SRR20326862, SRR14560308 (D. melanogaster); SRR33339778, SRR30855151, SRR14160649, SRR24948904, SRR24636232, SRR13159213, SRR8422221 (A. thaliana); SRR32903297, SRR31588239, SRR27369179, SRR28908270, SRR18855849, SRR24134730, SRR13981556 (M. musculus); and SRR32139880, SRR31785722, SRR25646397, SRR25732618, SRR22548603, SRR8357441, SRR7741229 (H. sapiens).

Complete CATS benchmark results generated in this study, including processed data and values underlying figures, have been deposited in Zenodo at https://doi.org/10.5281/zenodo.16837970 (34).

Research involving human participants, their data, or biological material

Policy information about studies with human participants or human data. See also policy information about sex, gender (identity/presentation), and sexual orientation and race, ethnicity and racism.

Reporting on sex and gender	Not applicable.
Reporting on race, ethnicity, or other socially relevant groupings	Not applicable.
Population characteristics	Not applicable.
Recruitment	Not applicable.
Ethics oversight	Not applicable.

Note that full information on the approval of the study protocol must also be provided in the manuscript.

Field-specific reporting

Please select the one below that is the best fit for your research. If you are not sure, read the appropriate sections before making your selection.

☒ Life sciences ☐ Behavioural & social sciences ☐ Ecological, evolutionary & environmental sciences

For a reference copy of the document with all sections, see nature.com/documents/nr-reporting-summary-flat.pdf

Life sciences study design

All studies must disclose on these points even when the disclosure is negative.

Sample size	Sample sizes in this study were not determined using formal statistical power calculations. Instead, they were chosen to ensure broad and systematic coverage of relevant experimental variables. The benchmark included 504 transcriptome assemblies from controlled simulations (126 RNA-seq libraries × 4 assemblers), 384 assemblies from realistic simulations (96 RNA-seq libraries × 4 assemblers), and 168 assemblies from 42 public RNA-seq libraries. Mutation analyses included 12 assemblies across four mutation levels and five distinct mutation types (240 assemblies with individual mutation types), as well as 12 assemblies subjected to six multiplicative mutation levels with triplicates (216 assemblies with multiplicative mutations). These sample sizes were defined to cover a wide range of species, sequencing depths, error rates, commonly used transcriptome assemblers, mutation classes, and mutation levels. The large number and diversity of datasets, along with replication across conditions, ensure robust and generalizable performance evaluation of the CATS framework.
Data exclusions	None of the analysed RNA-seq libraries or assemblies were excluded
Replication	The reproducibility of the findings was supported by extensive validation across multiple independent settings, including controlled simulations, realistic simulations, mutated assemblies, and public RNA-seq libraries. The tested datasets spanned a broad range of sequencing depths and error rates, and included six model species, four representative transcriptome assemblers, and five commonn mutation types.

Additionally, the code, processed benchmark data, underlying results, and fixed random seeds used for simulations are publicly available, further supporting reproducibility. Overall, replication was successful, as the main findings were consistently reproduced across diverse library and assembly conditions.

Randomization

Samples were not allocated into experimental groups through randomization, as this study is based on computational benchmarking rather than experimental treatment assignment. Instead, RNA-seq libraries were systematically simulated to represent predefined conditions. In controlled simulations, transcript coverage within each library was randomly sampled from predefined ranges. In realistic simulations, expressed transcripts and their coverage within each library were randomized across replicates (four replicates per sequencing depth and species combination) to capture variability. For mutation analyses, each mutation type was introduced into randomly selected subsets of transcripts, with mutations applied to random transcript regions. Public RNA-seq libraries were selected from the Sequence Read Archive by random sampling within each species to obtain a representative subset of datasets.

Blinding

Not applicable. This is a computational benchmarking study with no subjective measurements requiring blinding.

Reporting for specific materials, systems and methods

We require information from authors about some types of materials, experimental systems and methods used in many studies. Here, indicate whether each material, system or method listed is relevant to your study. If you are not sure if a list item applies to your research, read the appropriate section before selecting a response.

Materials & experimental systems

n/a	Involved in the study
☒	☐ Antibodies
☒	☐ Eukaryotic cell lines
☒	☐ Palaeontology and archaeology
☒	☐ Animals and other organisms
☒	☐ Clinical data
☒	☐ Dual use research of concern
☒	☐ Plants

Methods

n/a	Involved in the study
☒	☐ ChIP-seq
☒	☐ Flow cytometry
☒	☐ MRI-based neuroimaging

Plants

Seed stocks

Not applicable.

Novel plant genotypes

Not applicable.

Authentication

Not applicable.
