# Supplementary material for: Comprehensive assessment of transcriptome assembly quality using CATS
Source: Nat Commun. 2026 Apr 20;17:5419. doi: 10.1038/s41467-026-72171-8 (PMC13280353; doi:10.1038/s41467-026-72171-8)
Supplement: Supplementary file 3 — Transparent Peer Review file [file 41467_2026_72171_MOESM3_ESM.pdf]

nature portfolio

Peer Review File

Comprehensive Assessment of Transcriptome Assembly Quality using CATS

Corresponding Author: Professor Kristian Vlahoviček

This file contains all reviewer reports in order by version, followed by all author rebuttals in order by version.

Attachments originally included by the reviewers as part of their assessment can be found at the end of this file.

Version 0:

Reviewer comments:

Reviewer #1

(Remarks to the Author)
Please, see PDF attached.

General comments

The paper "Comprehensive Transcriptome Quality Assessment Using CATS: Reference-free and Reference-based Approaches" presents two new tools for reference-based and reference-free de novo transcriptome assembly quality assessment. While this problem was previously addressed by several software packages, the authors provide sufficient evidence to why their tool can be favoured over existing solutions. Thus, I expect this package to be a valuable addition to the scientific community in the future.

The GitHub page looks great and presents a full user manual. I was able to easily download, install and test both tools on the provided demo data and even on my own data. For me, usability is one of the key aspects in publishing scientific software. Both tools generate informative plots and reports that can be very useful for bioinformaticians working with transcriptome assemblies.

The manuscript itself is well-structured, concise and easy to follow. The claims are supported by a large number of plots and figures. The authors use a large collection of real and simulated datasets, perform transcriptome assemblies with various tools and examine a broad variety of different parameters. Moreover, they also provide some additional information on the performance of tested assemblers and how their tool changes this perspective, which can be of interest for a reader. Although the work looks solid and complete, below I provide a few suggestions that in my opinion may improve the software and the manuscript itself.

Major comments

1. Assemblers tested in this work were published at least 6 years ago, and based on their GitHub pages are not actively developed. At the same time, new tools attempt to incorporate long reads for transcriptome assemblies. These developments implement both hybrid [1, 2] and long-read-only approaches [1, 3, 4] . Moreover, as was claimed in the recent study [5], de novo assemblies expectedly show much lower quality on complex organisms compared to genome-based assemblies. Hence, there is an impression that de novo short-read transcriptome assembly "popularity" is already past its peak with overall interest shifting towards long reads or even towards genome-based transcript discovery with long reads, as long reads provide far more information on transcriptome diversity and alternative splicing. Hence, my question is: how hard would it be to incorporate long-read support into CATs-rb? This would make the software even more valuable and relevant for modern-day transcriptomics.

Minor comments

1. Since the README files are quite large, in the future the authors may want to consider creating structured multipage documentation, e.g. using mkdocs or wiki construction framework (not required for this particular revision).

2. Figures 2, 3 and 5. It took me a while to find the definitions at first, would it make sense to move them to the beginning of the caption?

References

1. Nip, K. M., Hafezqorani, S., Gagalova, K. K., Chiu, R., Yang, C., Warren, R. L., & Birol, I. (2023). Reference-free assembly of long-read transcriptome sequencing data with RNA-Bloom2. Nature Communications, 14(1), 2940.

2. Prjibelski, A. D., Puglia, G. D., Antipov, D., Bushmanova, E., Giordano, D., Mikheenko, A., ... & Lapidus, A. (2020). Extending rnaSPAdes functionality for hybrid transcriptome assembly. BMC bioinformatics, 21(Suppl 12), 302.

3. Petri, A. J., & Sahlin, K. (2023). isONform: reference-free transcriptome reconstruction from Oxford Nanopore data. Bioinformatics, 39(Supplement_1), i222-i231.

4. Petri, A. J., & Sahlin, K. (2025). De novo clustering of large long-read transcriptome datasets with isONclust3. Bioinformatics, 41(5), btaf207.

5. Pardo-Palacios, F. J., Wang, D., Reese, F., Diekhans, M., Carbonell-Sala, S., Williams, B., ... & Brooks, A. N. (2024). Systematic assessment of long-read RNA-seq methods for transcript identification and quantification. Nature methods, 21(7), 1349-1363.

(Remarks on code availability)
The GitHub page looks great and presents a full user manual. I was able to easily download, install and test both tools on the provided demo data and even on my own data. For me, usability is one of the key aspects in publishing scientific software. Both tools generate informative plots and reports that can be very useful for bioinformaticians working with transcriptome assemblies.

Reviewer #2

(Remarks to the Author)
This manuscript presents a pair of novel methods and associated software for evaluating de novo assemblies of short RNA-seq data. One method (CATS-rf) evaluates an assembly using only the reads from which it was derived and another (CATS-rb) evaluates an assembly based on its alignment to a reference genome (but with no reference annotation needed). The method is evaluated on both simluated and real RNA-seq data and compared with two other related methods: RSEM-EVAL and TransRate. With these experiments, CATS-rf compares favorably to the other methods, with a higher correlation with a ground-truth-based F-score measure defined by the authors.

Evaluation of de novo short-read RNA-seq assemblies without the ground-truth is a challenging problem, but is also one that is diminishing in significance as long-read technologies have matured and are replacing short-read technologies for the purpose of genome annotation. The manuscript does a good job at evaluating assemblies and tools across a range of species, sequencing parameters, and degrees of assembly errors. However, I have some major concerns about the evaluations and primary metrics used, as well as a lack of intuitive description and justification for the scores computed by the methods.

Major comments:

1. The primary metric used for evaluating the reference-free assembly scores is a mean F-score derived from reciprocal best-hits between assembled contigs and reference transcripts. In these scheme, there is no penalty for reference transcripts that are not recovered by the assembly and thus this metric does not capture the completeness of the assembly. An assembly with a single perfectly assembled transcript and no other contigs would receive a perfect score with this metric. This is not an appropriate metric for evaluating assembly scores from RSEM-EVAL and TransRate, which capture both the accuracy and completeness of an assembly given the reads from which it was derived.
2. Figure 1f: it is not appropriate to calculate a correlation between a reference-free score with a ground-truth score across assemblies from different read sets (and species), as reference-free scores such as from RSEM-EVAL and TransRate are meant for comparisons of assemblies from the same data, and their scores may not be comparable across different datasets.
3. Although simulation experiments are performed across different levels of read coverage (Fig 1e), it is not clear that the simulations capture the realistic distributions of read coverage within RNA-seq data where read coverage can vary by over 10^4 and most transcripts have low coverage. This wide range of coverage is what makes both de novo assembly and the evaluation of such assemblies particularly challenging. Simulations should be done with a realistic range of expression levels of transcripts within the same dataset.
4. Although the components of the CATS-rf and CATS-rb scores are defined in the methods, there is little intuition given for each equation, which make these scores challenging to intepret, even if the components are connected to some general concept of assembly quality. Some discussion of why the score components have these particular formulations is needed.
5. The components of the scores also involve a number of tunable parameters and it is not clear (A) what the values of these

parameters were or (B) how these values were chosen, and whether they were tuned specifically for evaluations that were done.
6. Much of the CATS-rf methodology appears similar to TransRate, yet there is no discussion of the connections between these methods. For example, TransRate also computes four component scores with similar meanings to those used by CATS-rf, and TransRate also takes the product of these component scores as part of a composite assembly score. Figure 1c is also analogous to the Figure 3B of the TransRate paper.

Minor comments:

7. I did not understand this sentence in the Discussion: "This reduction may result from the alignment-based filtering strategy applied to public datasets, which aimed to retain only transcripts represented in the reference transcriptome." I did not see many mention of such filtering in the methods or results. This filtering does not seem appropriate for evaluation of the de novo assembly tools - can this be filtering be removed?

(Remarks on code availability)

Version 1:

Reviewer comments:

Reviewer #1

(Remarks to the Author)
The authors addressed my main comment with sufficient information and fair arguments, as well as added a few details into the manuscript.

At this moment, I don't have any further comments about the manuscript and published software.
I hope the authors will find resources for maintaining this tool in the future.

Best regards
Andrey Prjibelski

(Remarks on code availability)
As stated earlier, the GitHub page looks great and presents a full user manual. I was able to easily download, install and test both tools on the provided demo data and even on my own data.
I would like the user manual to be converted to a multi-page documentation, but that's not a critical issue by any means.

Reviewer #2

(Remarks to the Author)
I appreciate the authors thorough revisions and responses to my prior comments. The revised manuscript is much improved. A have two remaining concerns:

1. I agree with the change to no longer evaluate the global assembly scores from RSEM-EVAL and TransRate using the mean F-score metric. However, there are two important points that I believe should be included in the introduction to put the methods and evaluations in the proper context:

1A. In the reference-free case, there are generally two forms of assembly scores: (1) transcript-level scores that characterize the quality of individual assembled transcripts and (2) global assembly scores which combine both the quality of the assembled transcripts and the completeness of the assembly. CATS-rf only addresses the first form of score, i.e., it allows one to identify transcripts that are well assembled vs. those that are not. RSEM-EVAL and TransRate offer the second form of score, and that is the focus of RSEM-EVAL.

1B. RSEM-EVAL's transcript-level scores are not actually measures of quality of the assembled transcripts, nor are they intended to be. These scores are referred to as "contig impact scores" and they measure how much each assembled transcript/contig contributes to the global assembly score, which takes into account both the quality and completeness of the assembly. Thus, comparing transcript-level quality scores to these contig impact scores is not really an "apples-to-apples" comparison.

2. In the response to my prior comment regarding the tunable parameters, the authors provided the results of a random grid search experiment in which the parameters were randomly varied and the effect on the mean of the score components was measured. These results should be included in the manuscript or at least in the supplement, as many readers and/or users will likely have concerns regarding the parameters of the method. Second, measuring the effect on the mean of the score components does not explicitly measure how the transcript-level scores reflect the quality of the transcripts. I suggest that the authors compute the correlation between transcript-level scores and F-scores as they have done in the main manuscript.

(Remarks on code availability)

Version 2:

Reviewer comments:

Reviewer #2

(Remarks to the Author)
The authors have satisfactorily addressed my prior comments.

(Remarks on code availability)

Open Access This Peer Review File is licensed under a Creative Commons Attribution 4.0 International License, which permits use, sharing, adaptation, distribution and reproduction in any medium or format, as long as you give appropriate credit to the original author(s) and the source, provide a link to the Creative Commons license, and indicate if changes were made.
In cases where reviewers are anonymous, credit should be given to 'Anonymous Referee' and the source.
The images or other third party material in this Peer Review File are included in the article's Creative Commons license, unless indicated otherwise in a credit line to the material. If material is not included in the article's Creative Commons license and your intended use is not permitted by statutory regulation or exceeds the permitted use, you will need to obtain permission directly from the copyright holder.
To view a copy of this license, visit https://creativecommons.org/licenses/by/4.0/

We sincerely thank the reviewers and editors for the time and effort invested in evaluating our manuscript. We have carefully considered all comments and have revised the manuscript accordingly. Below, we provide our detailed, point-by-point responses to each comment.

Reviewer 1:

General comments

The paper "Comprehensive Transcriptome Quality Assessment Using CATS: Reference-free and Reference-based Approaches" presents two new tools for reference-based and reference-free de novo transcriptome assembly quality assessment. While this problem was previously addressed by several software packages, the authors provide sufficient evidence to why their tool can be favoured over existing solutions. Thus, I expect this package to be a valuable addition to the scientific community in the future.

The GitHub page looks great and presents a full user manual. I was able to easily download, install and test both tools on the provided demo data and even on my own data. For me, usability is one of the key aspects in publishing scientific software. Both tools generate informative plots and reports that can be very useful for bioinformaticians working with transcriptome assemblies.

The manuscript itself is well-structured, concise and easy to follow. The claims are supported by a large number of plots and figures. The authors use a large collection of real and simulated datasets, perform transcriptome assemblies with various tools and examine a broad variety of different parameters. Moreover, they also provide some additional information on the performance of tested assemblers and how their tool changes this perspective, which can be of interest for a reader. Although the work looks solid and complete, below I provide a few suggestions that in my opinion may improve the software and the manuscript itself.

We thank the reviewer for the positive and encouraging assessment of our manuscript and tools. We appreciate that the reviewer took the time to install and test CATS-rf and CATS-rb on their own datasets. We have carefully considered all suggestions provided below and have addressed them in detail in the responses to the major and minor comments.

Major comments

1. Assemblers tested in this work were published at least 6 years ago, and based on their GitHub pages are not actively developed. At the same time, new tools attempt to incorporate long reads for transcriptome assemblies. These developments implement both hybrid [1, 2] and long-read-only approaches [1, 3, 4] . Moreover, as was claimed in the recent study [5], de novo assemblies expectedly show much lower quality on complex organisms compared to genome-based assemblies. Hence, there is an impression that de novo short-read transcriptome assembly "popularity" is already past its peak with overall interest shifting towards long reads or even towards genome-based transcript discovery with long reads, as long reads provide far more information on transcriptome diversity and alternative splicing. Hence, my question is: how hard would it be to incorporate long-read support into CATs-rb? This would make the software even more valuable and relevant for modern-day transcriptomics.

We fully agree that transcriptome analysis is evolving toward long-read and genome-guided approaches. Below we address the points raised.

1. Choice of Evaluated Assemblers

Our study incorporates rnaSPAdes and Trinity, two widely used short-read de novo transcriptome assemblers that continue to be considered state-of-the-art by multiple independent benchmarks (1,2). Importantly, both tools remain actively maintained: rnaSPAdes had its most recent release in May 2025 (3), and Trinity was last updated in July 2024 (4). While the other assemblers included in this study (IDBA-tran and SOAPdenovo-Trans) are indeed older, we intentionally selected them to provide a more comprehensive representation of commonly referenced de novo transcriptome assemblers. Taken together, this selection ensures that our benchmarks capture a relevant and representative spectrum of de novo assemblers used in the field.

2. Relevance of CATS-rb to Long-Read and Genome-Guided Assemblies

We fully agree that long-read transcriptomics, as well as genome-guided reconstruction, provide superior resolution of isoform diversity and alternative splicing. For this reason, we emphasize that CATS-rb already supports the evaluation of assemblies generated from short and long reads, as well as from both de novo and genome-guided approaches, as the tool operates solely on assembled transcript sequences. In the manuscript, however, we restricted benchmarking to short-read de novo assemblies to complement the CATS-rf benchmarks while keeping the length of the study manageable. To avoid any ambiguity, we have now explicitly clarified the compatibility of CATS-rb with long-read and genome-guided assemblies in the revised Discussion section (lines 292–293).

3. Long-Read Support in CATS-rf

At present, CATS-rf cannot evaluate long-read assemblies because its framework relies on short-read mapping and paired-end–derived metrics (e.g., local fidelity and integrity score components). Extending CATS-rf to long reads would require adopting appropriate mappers (e.g., minimap2) and replacing paired-read–dependent components. This would potentially enable new advantages, including isoform-level redundancy estimation and chimerism detection. Given the increasing popularity of long-read RNA-seq, we are actively exploring the strategies that would enable long-read support in future updates.

4. Continued Importance of Short-Read Assemblies

Although long-read sequencing is becoming increasingly important in transcriptomics, short-read RNA-seq continues to be the predominant technology due to its cost-effectiveness and established workflows. While differential expression analysis remains the primary application, short-read datasets are also widely used for transcript annotation and phylogenomics. (5,6). CATS-rf is relevant to all of these applications, particularly when reliable reference data are unavailable or incomplete. This is a common scenario for many taxa, for which de novo transcriptome assembly remains essential. As such, CATS-rf is well suited not only for current research but also for analyzing the vast amount of existing short-read RNA-seq data in public databases.

Minor comments

1. Since the README files are quite large, in the future the authors may want to consider creating structured multipage documentation, e.g. using mkdocs or wiki construction framework (not required for this particular revision).

Thank you for this suggestion. We agree that the GitHub README files are quite long and could be improved in terms of navigation. In response, we are currently working on a multi-page documentation site to better organize the information. This new documentation will be linked to the existing GitHub manuals in the near future.

2. Figures 2, 3 and 5. It took me a while to find the definitions at first, would it make sense to move them to the beginning of the caption?

To improve clarity, we have now labeled all abbreviations with "Abbreviations:" in Figures 2, 3, and 5. These updates are indicated in the revised manuscript (Figure legends; lines 952, 967, 997).

References:

1. Hölzer, M. & Marz, M. De novo transcriptome assembly: A comprehensive cross-species comparison of short-read RNA-Seq assemblers. *GigaScience* **8** (5), giz039 (2019).

2. Behera, S., Voshall, A., Moriyama, E. N. & Helder, I. N. Plant transcriptome assembly: Review and benchmarking. Bioinformatics Ch. 7 (Exon Publications, Brisbane, 2011).

3. SPAdes Github repository. Github. Available from https://github.com/ablab/spades (Accessed November 13, 2025).

4. Trinity Github repository. Github. Available from https://github.com/trinityrnaseq/trinityrnaseq (Accessed November 13, 2025).

5. Gong, B., Li, D., Łabaj, P. P., Pan, B., Novoradovskaya, N., Thierry-Mieg, D., et al. Targeted DNA-seq and RNA-seq of Reference Samples with Short-read and Long-read Sequencing. *Scientific Data* **11**, 892 (2024).

6. Wang, H., Xu, Y., Zhang, Z., Zhang, G., Tan, C. & Ye, L. Development and application of transcriptomics technologies in plant science. *Crop Design* **3** (2), 100057 (2024).

Reviewer 2:

General comments:

This manuscript presents a pair of novel methods and associated software for evaluating de novo assemblies of short RNA-seq data. One method (CATS-rf) evaluates an assembly using only the reads from which it was derived and another (CATS-rb) evaluates an assembly based on its alignment to a reference genome (but with no reference annotation needed). The method is evaluated on both simluated and real RNA-seq data and compared with two other related methods: RSEM-EVAL and TransRate. With these experiments, CATS-rf compares favorably to the other methods, with a higher correlation with a ground-truth-based F-score measure defined by the authors.

Evaluation of de novo short-read RNA-seq assemblies without the ground-truth is a challenging problem, but is also one that is diminishing in significance as long-read technologies have matured and are replacing short-read technologies for the purpose of genome annotation. The manuscript does a good job at evaluating assemblies and tools across a range of species, sequencing parameters, and degrees of assembly errors. However, I have some major concerns about the evaluations and primary metrics used, as well as a lack of intuitive description and justification for the scores computed by the methods.

We are grateful for the reviewer's constructive feedback regarding the benchmarking and methodological clarity. We have addressed all major concerns in detail and have revised the manuscript accordingly. Please find our responses to the comments below.

Major comments:

1. The primary metric used for evaluating the reference-free assembly scores is a mean F-score derived from reciprocal best-hits between assembled contigs and reference transcripts. In these scheme, there is no penalty for reference transcripts that are not recovered by the assembly and thus this metric does not capture the completeness of the assembly. An assembly with a single perfectly assembled transcript and no other contigs would receive a perfect score with this metric. This is not an appropriate metric for evaluating assembly scores from RSEM-EVAL and TransRate, which capture both the accuracy and completeness of an assembly given the reads from which it was derived.

We fully agree that mean transcript F-scores do not account for assembly completeness and therefore are not suitable for evaluating global assembly scores from RSEM-EVAL and TransRate. In response, we removed the stated analyses. Instead, we now correlate mean transcript F-scores with the mean transcript quality scores generated by each tool (Results; lines 142–147, 160–162, Discussion; line 295, Methods; lines 761–765, Fig. 2f, 2i). This change removes the confounding effect of completeness and allows a direct assessment of each tool's ability to predict the global accuracy of assembled transcripts.

Here, we also clarify why mean transcript F-scores were chosen for evaluating CATS-rf. By design, CATS-rf incorporates only transcript-level accuracy into its assembly score and deliberately excludes completeness measures derived from the input RNA-seq reads. Completeness measures implemented in other tools, such as the proportion of reads mapped to the assembly, are themselves influenced by assembly errors.

Incorporating these metrics into the final score would risk over-penalizing assemblies, as the same errors would be counted both in the transcript-level accuracy and indirectly through reduced read-mapping rates. Instead, CATS-rf reports the proportion of mapped reads as a separate metric, allowing users to evaluate completeness independently without conflating it with error-related penalties already reflected in the transcript-level metrics. Furthermore, completeness evaluations are instead provided through CATS-rb. Although the original manuscript discussed the interplay between assembly accuracy and completeness, we now further clarify these sections (Results; lines 261–264, Discussion; lines 368–369, 372–374), while also emphasizing the differing evaluation scopes of CATS-rf and CATS-rb (Results: lines 96–97, Discussion; lines 288–289, Methods; lines 584–586).

2. Figure 1f: it is not appropriate to calculate a correlation between a reference-free score with a ground-truth score across assemblies from different read sets (and species), as reference-free scores such as from RSEM-EVAL and TransRate are meant for comparisons of assemblies from the same data, and their scores may not be comparable across different datasets.

We agree with the reviewer that reference-free scores from RSEM-EVAL and TransRate were originally designed for comparing assemblies generated from the same read set. However, our intention in the assembly-level analyses was to evaluate whether the analysed tools exhibit consistent relationships with absolute accuracy of the assembled transcripts despite variation in biological and technical factors.

In practice, the final accuracy of any transcriptome assembly is shaped jointly by transcriptome complexity, library preparation protocols, sequencing depth, error profiles, read length, and assembler performance. Because these factors collectively determine the absolute quality of the resulting transcripts, we sought to test whether reference-free tools can capture this underlying accuracy across heterogeneous assemblies. We have now clarified this rationale explicitly in the revised manuscript (Results; lines 143–145, Discussion; lines 320–325).

Although not specifically requested, we have also strengthened the transcript-level analyses to avoid potential confounding from pooling transcripts across libraries. Instead of computing pooled correlations, we now calculate correlations between reference-free transcript scores and F-scores within each assembly, and summarize these values across assemblies (Results; lines 126–134, 156–158, Methods; lines 761–762, Fig. 2d, Figure legends; lines 941–946, 948–951). We applied the same per-assembly approach to score-component intercorrelations (Results; lines 116–121, Fig. 2c, Figure legends; lines 940–941). This analysis preserves assembly-level structure and removes the confounding of transcript-level accuracy evaluation by library-specific effects.

Taken together, the transcript- and assembly-level results show that CATS-rf provides accurate assessments of transcript quality both within individual assemblies and across heterogeneous datasets. The latter finding is particularly notable: in contrast to existing reference-free tools, CATS-rf maintains a reliable relationship with the absolute accuracy of assembled transcripts even when assemblies differ in species, library characteristics, and sequencing conditions. As a result, CATS-rf offers improved reliability for comparing assemblies derived from different libraries, a task for which existing methods show notably weaker performance.

3. Although simulation experiments are performed across different levels of read coverage (Fig 1e), it is not clear that the simulations capture the realistic distributions of read coverage within RNA-seq data where read coverage can vary by over 10^4 and most transcripts have low coverage. This wide range of coverage is what makes both de novo assembly and the evaluation of such assemblies particularly challenging. Simulations should be done with a realistic range of expression levels of transcripts within the same dataset.

We appreciate the reviewer's comment and agree that real RNA-seq datasets typically exhibit substantial variability in transcript-level coverage. Our initial simulation framework was intentionally designed as an idealized setting in which intra-library coverage was relatively uniform and all reference transcripts were treated as expressed. This controlled design allowed us to vary transcriptome complexity (species), coverage, sequencing error rate, and assembler choice in an orthogonal manner, thereby isolating the contribution of each factor to CATS scoring without confounding from heterogeneous expression levels or unexpressed transcripts. It also provided a consistent basis for benchmarking across a broad spectrum of assembly conditions. Finally, the controlled setting enabled a detailed examination of how specific mutation types influence scoring in transcripts with sufficient coverage, as well as a systematic evaluation of relative CATS-rb assembly scores under progressively reduced assembly quality. Such mechanistic analyses would not be feasible in realistic RNA-seq datasets, where multifold variation in coverage and a limited set of expressed transcripts preclude the orthogonality required for these comparisons.

We fully acknowledge, however, that this controlled framework does not capture the wide range of expression and coverage observed in real RNA-seq data. To address this limitation, the original manuscript included analyses of assemblies derived from public RNA-seq libraries. While these datasets allow us to examine performance under realistic conditions, the absence of ground-truth transcript sets (as discussed in Reviewer Point 7 and in the manuscript's Discussion) constrains the interpretability of those results.

To strengthen the study, the revised manuscript now incorporates a new set of realistically simulated libraries that exhibit intra-library coverage variation on the order of 10^4–10^5-fold and a realistic proportion of expressed transcripts. These datasets consist of 96 libraries spanning four sequencing depth levels and include the same six species used in both the controlled simulations and the public RNA-seq analyses. Each species–coverage combination was generated in four independent replicates. Benchmarking results obtained from these datasets have been added to the manuscript (Results; lines 149–162, 247–258, Discussion; lines 300–304, 333–334, 358, 390–392, 394–396, Fig. 2g, 2h, 2i, 5d, 5e, Figure legends; lines 947–951, 989–991, Supplementary Fig. 7) We also provide a detailed description of the simulation procedure and benchmarking (Methods; lines 687–705, 754–756, 778–781, 789–792) and report the transcript-level coverage distributions for these realistic simulations (Supplementary Fig. 11). Importantly, the results from realistic simulations are consistent with those from controlled and public datasets, further supporting our conclusions.

The revised manuscript therefore evaluates CATS across three progressively complex settings: controlled simulations, realistic simulations, and public RNA-seq assemblies. Several sections have been updated to clarify the rationale behind the controlled simulations (Results; lines 102–103, 105–107, 122–124, 138–140, 175–177, 227–229, 238, 240–241, 245–246), Discussion; lines 296–300, 336–340, 358, 389, 393–397,

Methods; lines 674–676, 681–682, 778–781, 785–786, Figure legends; lines 938, 986–987, 993–994) and to reflect the updated total number of analysed assemblies (Abstract; line 22, Methods; lines 714–715). To strengthen this narrative, we also added two new supplementary figures (Supplementary Figs. 1 and 2) to further analyze CATS-rf scoring in the context of assembly features examined within the controlled dataset.

4. Although the components of the CATS-rf and CATS-rb scores are defined in the methods, there is little intuition given for each equation, which make these scores challenging to intepret, even if the components are connected to some general concept of assembly quality. Some discussion of why the score components have these particular formulations is needed.

Thank you for this comment. We agree that providing clearer intuition for the formulation of each score component would improve the interpretability of the definitions. In response, we have substantially expanded the Methods section to explicitly articulate the rationale underlying each equation and to clarify how the structure of each score component reflects specific aspects of transcript quality (lines 442–445, 452–453, 459, 461–465, 475–478, 482–485, 492–499, 510–512, 537–539, 541, 546–548, 554–556, 578–582, 584–586).

5. The components of the scores also involve a number of tunable parameters and it is not clear (A) what the values of these parameters were or (B) how these values were chosen, and whether they were tuned specifically for evaluations that were done.

We agree that the default values of the tunable parameters require clearer explanation. Because RNA-seq libraries differ substantially in their biological and technical characteristics, optimizing these parameters in a fully data-driven manner would require a large number of representative datasets to ensure generalizable performance. The computational time and resources required for such a large-scale tuning approach were infeasible. For this reason, the parameters were instead defined as moderate, biologically reasonable values rather than tuned to any specific evaluation dataset. The default parameter values and their justification are now detailed in Supplementary Table 2, with the addition referenced in the Methods section (lines 436–439).

To evaluate the impact of parameter variation on CATS-rf score components, we performed a random grid-search analysis using 16 assemblies from realistic simulations (rnaSPAdes assemblies of *S. cerevisiae*, *C. elegans*, *D. melanogaster*, and *A. thaliana*, coverage 20×, 40×, 60×, and 80×). As shown in Response Fig. 1, we systematically varied each tunable parameter (x-axis) and measured its effect on the corresponding score components. The results show that moderate parameter variation produces limited changes in the associated score components, indicating that the method is robust to varying parameter settings.

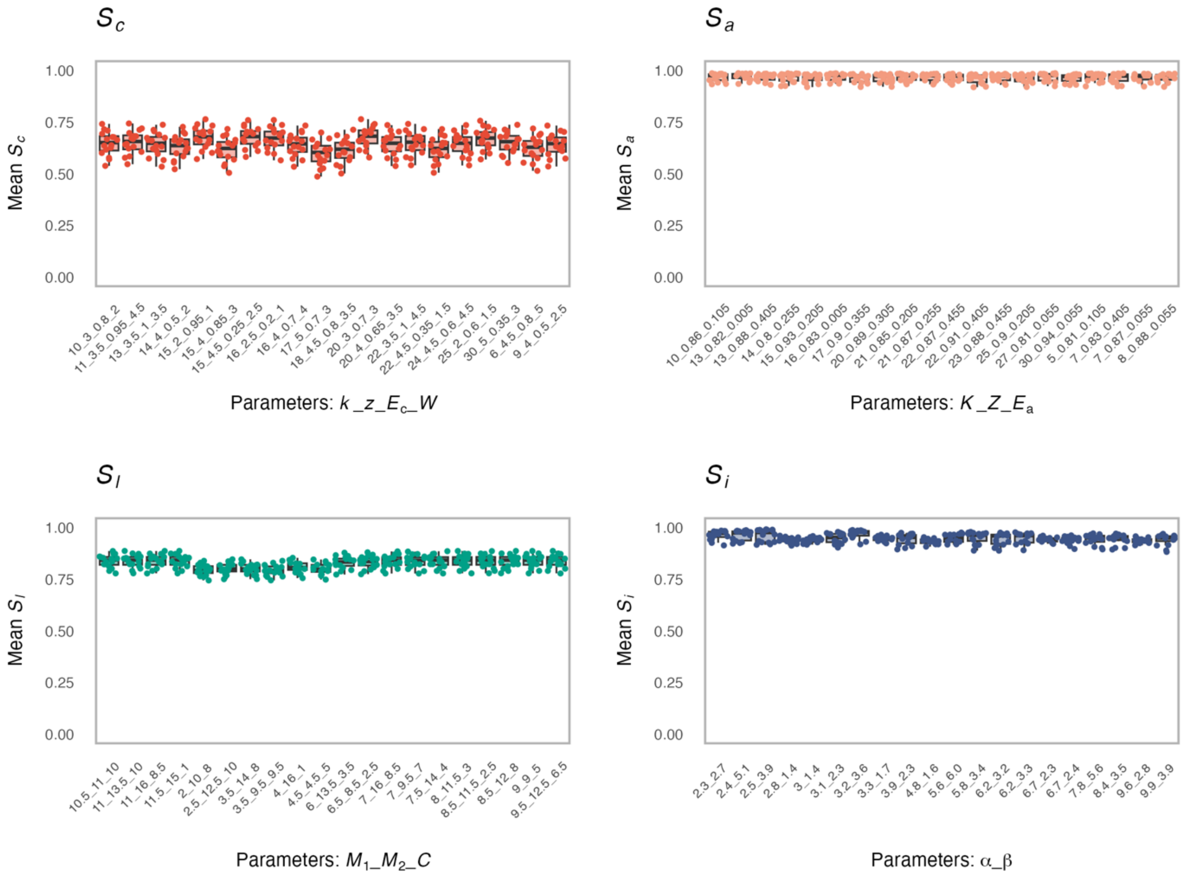

Response Figure 1. Distribution of mean score components across varying CATS-rf scoring parameters. The parameters were varied using a random grid-search approach, as indicated on the x-axis. For details on the evaluated parameters consult Supplementary Table 2. S_c = coverage component, S_a = accuracy component, S_l = local fidelity component, S_i = integrity component, k = rolling window length for LCR detection, z = mean rolling window coverage threshold for LCR definition, E_c = LCR extension penalty, W = base coverage weight, K = rolling window length for LAR detection, Z = mean rolling window accuracy threshold, E_a = LAR extension penalty, M_1 = lower multiplicative factor, M_2 = higher multiplicative factor, C = correction factor, α = compression factor 1, β = compression factor 2.

6. Much of the CATS-rf methodology appears similar to TransRate, yet there is no discussion of the connections between these methods. For example, TransRate also computes four component scores with similar meanings to those used by CATS-rf, and TransRate also takes the product of these component scores as part of a composite assembly score. Figure 1c is also analogous to the Figure 3B of the TransRate paper.

We appreciate the reviewer’s observation regarding the conceptual similarities between CATS-rf and TransRate. While both methods indeed make use of read-mapping evidence to evaluate transcript quality, the underlying methodologies differ substantially. These differences include the read-mapping strategy, the approach to multimapped read assignment, the handling of paired-end information, and the

formulation and scope of transcript score components. In addition, the definitions of assembly-level composite scores are distinct between the two approaches.

CATS-rf also goes beyond TransRate by providing a comprehensive set of detailed metrics and diagnostic plots, enabling users to more thoroughly assess and troubleshoot their assemblies. To clarify these points for readers, we have expanded the Discussion section to explicitly compare CATS-rf and TransRate (lines 326–329). We have also included a new Supplementary Table 1 that offers a detailed, side-by-side comparison of the two methods.

Minor comments:

7. I did not understand this sentence in the Discussion: "This reduction may result from the alignment-based filtering strategy applied to public datasets, which aimed to retain only transcripts represented in the reference transcriptome." I did not see many mention of such filtering in the methods or results. This filtering does not seem appropriate for evaluation of the de novo assembly tools - can this be filtering be removed?

Thank you for this comment. The alignment-based filtering strategy is indeed described in the original manuscript (Results, lines 166–167; Methods, lines 756–758). In brief, the filtering involved retaining only those assembled transcripts represented in the reference transcriptome.

When benchmarking on public RNA-seq datasets, we encountered a substantial issue: a considerable fraction of assembled transcripts had no identifiable counterpart in the reference transcriptome. This mismatch can arise due to multiple technical and biological factors, including contamination, unannotated non-coding transcripts, novel isoforms, in vivo chimeras, and transcript degradation. If such transcripts were included in the evaluation, CRB-BLAST would necessarily assign them an incorrect F-score due to the lack of the corresponding reference. This would artificially distort the correlation between transcript quality scores and F-scores, penalizing the assemblies for reasons unrelated to accuracy.

To mitigate the lack of ground-truth transcriptome assemblies in public datasets, we restricted the benchmark to transcripts that were represented in the reference. This was done by aligning the assembled transcripts to the corresponding reference transcriptome using BLAT and retaining only alignments with F-score higher than 0.5. We acknowledge that this approach still creates bias by excluding low-quality transcripts. However, the bias introduced without such filtering is substantially greater, as the evaluation would be greatly confounded by transcripts without a reference counterpart.

We have further clarified this limitation and its implications in the revised version of the manuscript (Discussion; lines 295–304, Methods; lines 756–757). Notably, the realistically simulated datasets added in this revision help bridge the gap between controlled simulations and public-data benchmarking, providing a setting in which all true transcripts are known and can be evaluated without filtering.

We sincerely thank the reviewers and editors for the time and effort invested in the second revision of our manuscript. We have considered all comments and have revised the manuscript accordingly. Below, we provide our detailed responses to each comment.

Reviewer 1:

The authors addressed my main comment with sufficient information and fair arguments, as well as added a few details into the manuscript.

At this moment, I don't have any further comments about the manuscript and published software.

I hope the authors will find resources for maintaining this tool in the future.

As stated earlier, the GitHub page looks great and presents a full user manual. I was able to easily download, install and test both tools on the provided demo data and even on my own data. I would like the user manual to be converted to a multi-page documentation, but that's not a critical issue by any means.

We thank the reviewer for their positive evaluation of the revised manuscript and for their constructive feedback during the review process. We are pleased that the revisions adequately addressed the reviewer’s concerns. We also appreciate the reviewer’s encouragement regarding future maintenance of the software and are committed to ensuring its continued availability, user accessibility, and support.

Reviewer 2:

I appreciate the authors thorough revisions and responses to my prior comments. The revised manuscript is much improved. A have two remaining concerns

We are grateful for the reviewer’s feedback on our first revision. We have addressed both comments and have revised the manuscript accordingly. Please find our responses to the comments below

1. I agree with the change to no longer evaluate the global assembly scores from RSEM-EVAL and TransRate using the mean F-score metric. However, there are two important points that I believe should be included in the introduction to put the methods and evaluations in the proper context:

1A. In the reference-free case, there are generally two forms of assembly scores: (1) transcript-level scores that characterize the quality of individual assembled transcripts and (2) global assembly scores which combine both the quality of the assembled transcripts and the completeness of the assembly. CATS-rf only addresses the first form of score, i.e., it allows one to identify transcripts that are well assembled vs. those that are not. RSEM-EVAL and TransRate offer the second form of score, and that is the focus of RSEM-EVAL.

1B. RSEM-EVAL's transcript-level scores are not actually measures of quality of the assembled transcripts, nor are they intended to be. These scores are referred to as "contig impact scores" and they measure how much each assembled transcript/contig contributes to the global assembly score, which takes into account both the quality and completeness of the assembly. Thus, comparing transcript-level quality scores to these contig impact scores is not really an "apples-to-apples" comparison.

We agree with these remarks and have incorporated them into multiple sections of the revised manuscript to better contextualize the compared methods. The Introduction section now contains a more detailed description of RSEM-EVAL (lines 47–52; 56–57) and TransRate (lines 62–66) scoring schemes. The Results section has been revised to more clearly frame the scope and intent of each tool prior to presenting comparative analyses (lines 103–105; 131–133). Finally, the Discussion section has been updated to interpret the benchmark results in light of the stated methodological differences (lines 326–331).

2. In the response to my prior comment regarding the tunable parameters, the authors provided the results of a random grid search experiment in which the parameters were randomly varied and the effect on the mean of the score components was measured. These results should be included in the manuscript or at least in the supplement, as many readers and/or users will likely have concerns regarding the parameters of the method. Second, measuring the effect on the mean of the score components does not explicitly measure how the transcript-level scores reflect the quality of the transcripts. I suggest that the authors compute the correlation between transcript-level scores and F-scores as they have done in the main manuscript.

We agree that evaluating the correlation between transcript-level scores and F-scores provides a more appropriate and direct assessment of the robustness of CATS-rf parameters, as it measures how accurately the method reflects transcript quality. In response, we have performed the requested analysis and added it to the manuscript as Supplementary Figure 11. In the analysis, we compute the correlation between CATS-rf transcript-level score components and F-scores across the full range of parameters explored in the random grid search, alongside the default parameters. The results demonstrate limited variation in correlation values across parameter settings, indicating that the performance of CATS-rf is robust to reasonable changes in its tunable parameters. We have also included a detailed description of the random grid search and correlation analysis in the Supplementary Methods, and we now explicitly reference this new analysis in the main text (Methods; lines 442–444). This addition provides greater transparency regarding parameter sensitivity and addresses potential concerns from users regarding parameter selection.

General comments

The paper "Comprehensive Transcriptome Quality Assessment Using CATS: Reference-free and Reference-based Approaches" presents two new tools for reference-based and reference-free de novo transcriptome assembly quality assessment. While this problem was previously addressed by several software packages, the authors provide sufficient evidence to why their tool can be favoured over existing solutions. Thus, I expect this package to be a valuable addition to the scientific community in the future.

The GitHub page looks great and presents a full user manual. I was able to easily download, install and test both tools on the provided demo data and even on my own data. For me, usability is one of the key aspects in publishing scientific software. Both tools generate informative plots and reports that can be very useful for bioinformaticians working with transcriptome assemblies.

The manuscript itself is well-structured, concise and easy to follow. The claims are supported by a large number of plots and figures. The authors use a large collection of real and simulated datasets, perform transcriptome assemblies with various tools and examine a broad variety of different parameters. Moreover, they also provide some additional information on the performance of tested assemblers and how their tool changes this perspective, which can be of interest for a reader. Although the work looks solid and complete, below I provide a few suggestions that in my opinion may improve the software and the manuscript itself.

Major comments

1. Assemblers tested in this work were published at least 6 years ago, and based on their GitHub pages are not actively developed. At the same time, new tools attempt to incorporate long reads for transcriptome assemblies. These developments implement both hybrid [1, 2] and long-read-only approaches [1, 3, 4] . Moreover, as was claimed in the recent study [5], de novo assemblies expectedly show much lower quality on complex organisms compared to genome-based assemblies. Hence, there is an impression that de novo short-read transcriptome assembly "popularity" is already past its peak with overall interest shifting towards long reads or even towards genome-based transcript discovery with long reads, as long reads provide far more information on transcriptome diversity and alternative splicing. Hence, my question is: how hard would it be to incorporate long-read support into CATs-rb? This would make the software even more valuable and relevant for modern-day transcriptomics.

Minor comments

1. Since the README files are quite large, in the future the authors may want to consider creating structured multipage documentation, e.g. using mkdocs or wiki construction framework (not required for this particular revision).
2. Figures 2, 3 and 5. It took me a while to find the definitions at first, would it make sense to move them to the beginning of the caption?

References

1. Nip, K. M., Hafezqorani, S., Gagalova, K. K., Chiu, R., Yang, C., Warren, R. L., & Birol, I. (2023). Reference-free assembly of long-read transcriptome sequencing data with RNA-Bloom2. Nature Communications, 14(1), 2940.
2. Prjibelski, A. D., Puglia, G. D., Antipov, D., Bushmanova, E., Giordano, D., Mikheenko, A., ... & Lapidus, A. (2020). Extending rnaSPAdes functionality for hybrid transcriptome assembly. BMC bioinformatics, 21(Suppl 12), 302.
3. Petri, A. J., & Sahlin, K. (2023). isONform: reference-free transcriptome reconstruction from Oxford Nanopore data. Bioinformatics, 39(Supplement_1), i222-i231.
4. Petri, A. J., & Sahlin, K. (2025). De novo clustering of large long-read transcriptome datasets with isONclust3. Bioinformatics, 41(5), btaf207.
5. Pardo-Palacios, F. J., Wang, D., Reese, F., Diekhans, M., Carbonell-Sala, S., Williams, B., ... & Brooks, A. N. (2024). Systematic assessment of long-read RNA-seq methods for transcript identification and quantification. Nature methods, 21(7), 1349-1363.
